# Intermolecular ‘cross-torque’: the *N^4^*-cytosine propargyl residue is rotated to the ‘CH’-edge as a result of Watson–Crick interaction

**DOI:** 10.1093/nar/gkv285

**Published:** 2015-04-30

**Authors:** Olwen Domingo, Isabell Hellmuth, Andres Jäschke, Christoph Kreutz, Mark Helm

**Affiliations:** 1 Department of Pharmaceutical Chemistry, Institute of Pharmacy and Biochemistry, Johannes Gutenberg University, 55128 Mainz, Rhineland-Palatinate, Germany; 2 Institute of Pharmacy and Molecular Biotechnology, Heidelberg University, 69120 Heidelberg, Baden-Wuerttemberg, Germany; 3 Institute of Organic Chemistry, University of Innsbruck, 6020 Innsbruck, Tyrol, Austria

## Abstract

Propargyl groups are attractive functional groups for labeling purposes, as they allow CuAAC-mediated bioconjugation. Their size minimally exceeds that of a methyl group, the latter being frequent in natural nucleotide modifications. To understand under which circumstances propargyl-containing oligodeoxynucleotides preserve base pairing, we focused on the exocyclic amine of cytidine. Residues attached to the exocyclic *N4* may orient away from or toward the Watson–Crick face, ensuing dramatic alteration of base pairing properties. ROESY-NMR experiments suggest a uniform orientation toward the Watson–Crick face of *N^4^*-propargyl residues in derivatives of both deoxycytidine and 5-methyl-deoxycytidine. In oligodeoxynucleotides, however, UV-melting indicated that *N^4^*-propargyl-deoxycytidine undergoes standard base pairing. This implies a rotation of the propargyl moiety toward the ‘CH’-edge as a result of base pairing on the Watson–Crick face. In oligonucleotides containing the corresponding 5-methyl-deoxycytidine derivative, dramatically reduced melting temperatures indicate impaired Watson–Crick base pairing. This was attributed to a steric clash of the propargyl moiety with the 5-methyl group, which prevents back rotation to the ‘CH’-edge, consequently preventing Watson–Crick geometry. Our results emphasize the tendency of an opposing nucleic acid strand to mechanically rotate single *N^4^*-substituents to make way for Watson–Crick base pairing, providing no steric hindrance is present on the ‘CH’-edge.

## INTRODUCTION

The chemical modification of nucleic acid monomers has become common practice in a variety of research fields for gaining access to artificially enhanced DNA and RNA properties. In particular, [3+2] cycloaddition reactions between azides and alkynes (CuAAC and SPAAC), together with other forms of click reactions, are amongst the favorites of the bioconjugation reaction types and require the incorporation of functionalities onto the nucleic acid monomers (1–6).

The exocyclic amine groups in nucleobases are interesting targets for the attachment of small groups, such as terminal alkynes. Terminal alkynes, because of their small size, can be expected to serve as a tag, which marks a specific point for later bioconjugation via click labeling, while exacting minimal perturbance until that time. Since functionalization is likely to affect the hydrogen bonding edges of the nucleoside, it is important to consider a potential influence on base pairing, and, as a consequence, also on helical stability. Taking a clue from naturally occurring modifications of exocyclic amines, we observed that *N^4^*-methylcytidine (m^4^C) and *N^6^*-methyladenosine (m^6^A) occur in Watson–Crick helices of both DNA and RNA (7–11), where presumably the methyl group would be rotated toward the Hoogsteen edge/‘CH’-edge, leaving the second hydrogen on the Watson–Crick free to interaction in hydrogen bonding. By generating a crystal structure of a hexameric duplex, Cervi *et al*. (12) have shown that this is indeed the case for m^4^C. In contrast, double methylation in *N^4^*,*N^4^*-dimethylcytidine (${\rm m}_2^4$C), *N^6^*,*N^6^*-dimethyladenosine (${\rm m}_2^6$A) and *N^2^*,*N^2^*-dimethylguanosine (${\rm m}_2^2$G) is incompatible with standard Watson–Crick pairing, although *N^2^*,*N^2^*-dimethylguanosine (${\rm m}_2^2$G) in certain RNAs is thought to instead form a ‘wobble’-pair with uridine (13), which avoids implication of the exocyclic *N2* in hydrogen bonding. More bulky, albeit single modifications of the exocyclic *N6* of adenosine (t^6^A, i^6^A), occur in single stranded regions of tRNA (14), as do single methylations of *N1*/*N3* on the Watson–Crick face (m^1^A, m^1^G, m^3^C, m^3^U), which are also incompatible with standard base pairing (15).

A previous effort to introduce clickable units onto nucleosides was focused on *N^2^*-propargylated guanosine in RNA (16). Earlier reports on alkylation of the exocyclic guanosine amine had suggested minimal impact on duplex stability for single substitutions, and had also reported that significant differences in melting temperature of modified duplexes were only evident upon a double methylation (17–19). In agreement with this finding, our results showed that mono-labeling on the sugar edge of guanosine via its exocyclic amine had little impact on structure and RNAi activity of an siRNA labeled on the 5′ of the antisense strand (16). However, data on the impact of propargyl groups on exocyclic amines inside helices are still amiss.

**Figure 1.**
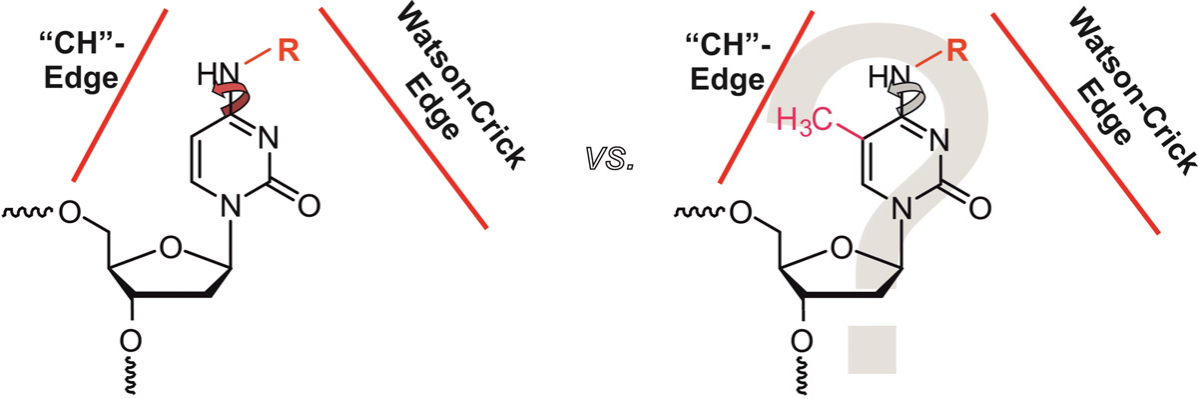
Hypothetical orientation of *N^4^*-substituents. A single modification (R) on the exocyclic amine of cytosine may, in principle, orient toward the Watson–Crick edge, or be twisted toward the ‘CH’-edge, thus enabling normal Watson–Crick base pairing within a double helix. For an additional methyl group on position 5 of the pyrimidine ring, a steric clash might impede this feature.

Literature on substituents on the cytidinic exocyclic amine suggested the *N4* of cytidines as a viable target for propargylation. While various reports do not show any indications of destabilization of nucleic acid duplexes containing *N^4^*-methylcytidine (20–23), investigations on the nucleoside level by the laboratories of von Hippel (24) and Becker (25), respectively, described a preferred orientation of the exocyclic amine substituent in the single nucleoside toward the Watson–Crick edge, mainly due to restricted rotation around the *C^4^*–*N^4^* bond. In their studies, von Hippel (24) and Becker (25), however, have shown that a methyl group on the exocyclic amine of cytidine has no particular effect on Watson–Crick base pairing in the context of an oligodeoxynucleotide. This suggests that a small *N^4^*-substituent will preferentially orient toward the Watson–Crick edge, but that hybridization into duplexes entails enough free energy to compensate for the minor penalty required to reorient the substituent toward the Hoogsteen edge (Figure 1).

Hence, we decided to investigate the preferred orientation of an *N^4^*-propargyl group in cytidines. Our approach to this problem was based on the notion that no naturally occurring double modifications of cytidine are known that combine additions to both the ‘CH’-edge and the exocyclic *N4*. Even synthetic work on dual modifications of this type is, to our knowledge, restricted to Carell's 5-methyl-2′-deoxycytidine-*O*-allylhydroxylamine adduct (26), which displayed a highly interesting property, namely a steric repulsion of the 5-methyl group and the flexible ligand at the 4-position. This allowed detection of the 5-methyl group by virtue of restricted rotation around the *C^4^–N^4^* bond in the pyrimidine base, leading to a preferential *cis*-conformation of the substitution on *N4*, thus causing interference with Watson–Crick base pairing within a sequence context.

Since the same effect would be expected of our *N^4^*-propargyl substituent, we conducted a comparative study on the nucleoside level by NMR, and in an oligonucleotide context by UV-melting curves on modified cytidines with and without 5-CH_3_-modification. Our results show that the 5-CH_3_-modification destabilizes both A- and B-type helices when present in combination with even a single substituent on position *N4* of cytidine. In the absence of that methyl group however, the propargyl group is well tolerated, thus identifying a position for DNA tagging with minimal structural interference.

## MATERIALS AND METHODS

Chemical reagents and solvents for monomer and oligonucleotide syntheses and purification were obtained from Sigma–Aldrich (Munich, Germany). Thin layer chromatography was done on pre-coated Polygram Sil G/UV_254_ silica gel plates, obtained from Macherey-Nagel (Dueren, Germany). Column chromatography was performed on either silica gel 60 (mesh 230–400 mesh) or neutral aluminium oxide 90 (70–230 mesh) from Merck (Darmstadt, Germany). Mass spectra of the modified monomers were either recorded on a Finningan MAT 95 or a Micromass LCT mass spectrometer. A Bruker AC 300 MHz was used for measuring ^1^H and ^13^C NMRs. ^31^P NMRs of the final phosphoramidites were measured on an Avance III HD 400 MHz. 2D phase sensitive ROESY NMR measurements of the deprotected derivatives were done on a Bruker Avance 300 MHz, with a mixing time of 250 ms and relaxation delay of 2 s. Spectral width was 10 ppm in both dimensions. An AVATAR 330 FT-IR (Thermo Nicolet) was used for infrared measurements.

An Agilent 1100 series was used for oligonucleotide purification on a DNAPac PA200 column (250 × 4 mm), eluting with a gradient system of increasing concentrations of NaClO_4_ in Tris buffer (pH 8). Details of HPLC purification can be found in the supporting information section. Masses of the final oligonucleotides were confirmed on a Bruker BIFLEX III by means of matrix-assisted laser desorption/ionization time-of flight (MALDI-TOF) mass spectrometry.

### Nucleoside modification and phosphoramidite synthesis

**Scheme 1.**
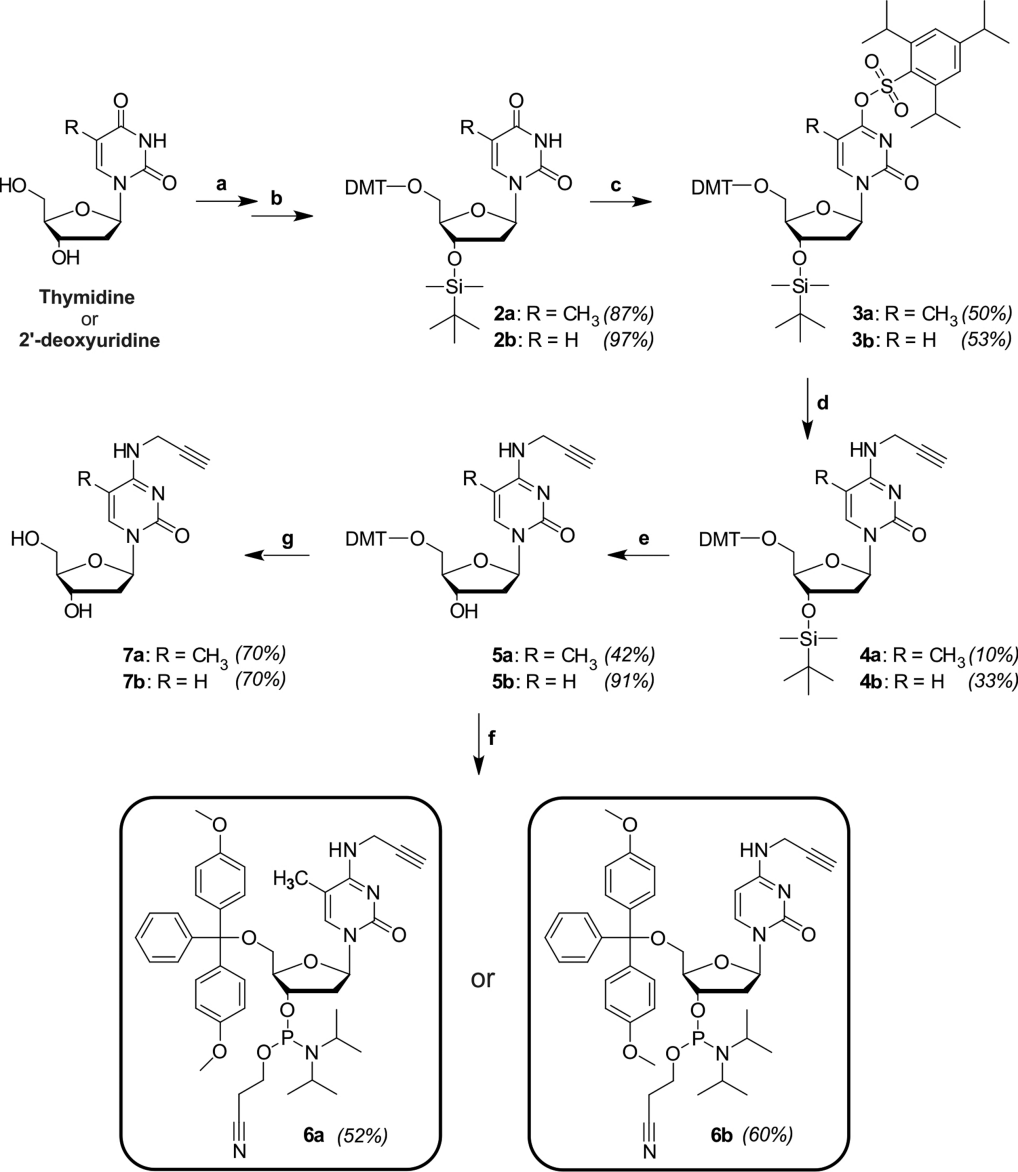
Synthesis of the respective phosphoramidite building blocks, **6a** and **6b**, together with the deprotected modified nucleosides for NMR analysis, **7a** and **7b**. Reagents and solvents: (**a**) DMT-Cl (4,4′-dimethoxytrityl chloride)/pyridine, (**b**) TBDMS-Cl (*tert*-butyldimethylsilyl chloride)/THF, (**c**) TPS-Cl (triisopropylbenzenesulfonyl chloride)/DCM, (**d**) propargylamine/dioxane, (**e**) TBAF (tetrabutylammonium fluoride)/THF, (**f**) CEP-Cl (2-cyanoethyl-*N*, *N*-diisopropylaminechlorophosphoramidite)/DCM and (**g**) TFA (trifluoroacetic acid)/DCM.

Additional details to the synthesis procedures can be found in the supplementary section. For the synthesis of both the 5-CH_3_ containing and the non-methyl containing propargylated cytidine derivatives, the same reaction route was followed starting from either thymidine or 2′-deoxyuridine, respectively. The synthesis procedure toward the phosphoramidite building blocks is depicted in Scheme 1 below.

#### Tritylation reaction

Starting from a pyridine solution of thymidine and 2′-deoxyuridine respectively, 0.5 equiv 4-dimethylamino pyridine (DMAP) was added, to which a solution of dimethoxytrityl chloride (DMT-Cl) in pyridine was slowly dropped. Following a reaction time of ∼12 h, methanol was added to quench the reaction and the crude product was purified by means of column chromatography. The tritylated products, **1a** and **2a**, were obtained in 94% and 83% yield, respectively.

#### TBDMS-protection

The respective tritylated compounds, **1a** and **2a**, were treated with 4.4 equiv imidazole and 4 equiv *tert-*butyldimethylsilyl chloride (TBDMS-Cl) in dichloromethane at ambient temperature. After extraction from NaHCO_3_ and brine, the products were isolated in 87% and 97% yield.

#### Incorporation of the sulfonyl ester

The deoxyribose-protected products were both treated with DMAP and triethylamine in dichloromethane, after which 1.2 equiv triisopropylbenzenesulfonyl chloride (TPS-Cl) was added. The reaction was allowed to stir at ambient temperature overnight under an argon atmosphere. The reaction mixtures were dried *in vacuo* and column chromatography resulted in the pure sulfonyl esters, **3a** and **3b**, in 50% and 25% yield, respectively.

#### Aminolysis with propargylamine

Twenty equivalents of propargylamine was added to a solution of the respective sulfonyl esters in dry dioxane. The reaction was allowed to proceed at ambient temperature overnight. The products were purified by means of column chromatography and resulted in 10% and 33% yield for **4a** and **4b**.

#### Desilylation

**4a** and **4b** were dissolved in anhydrous tetrahydrofuran and placed under argon. 1.6 equiv tetrabutylammonium fluoride (TBAF) was added to each solution. The desilylation reaction was complete after 1 h at ambient temperature. Ethyl acetate was added to the mixture and extraction from NaHCO_3_ was performed. Column chromatography with aluminium oxide resulted in products **5a** and **5b** in yields of 42% and 91%, respectively.

#### Phosphoramidite synthesis

The phosphoramidites were formed by treating compounds **5a** and **5b** with 5 equiv *N*,*N-*diisopropylethylamine and cyanoethyl-*N*,*N*-diisopropylaminechlorophosphoramidite (CEP-Cl) in anhydrous dichloromethane and under an argon atmosphere. The reaction mixtures were stirred at ambient temperature for 2 h, after which the solutions were extracted with 5% aqueous NaHCO_3_. After column chromatography, the products **7a** and **7b** were isolated in 52% and 60% yield, respectively.

#### Detritylation for ROESY NMR experiments

Compounds **5a** and **5b** were detritylated by treatment with 1.5 equiv trifluoroacetic acid (TFA). Both detritylated compounds (**7a/7b**) were collected in 70% yield after purification by column chromatography.

#### ROESY-NMR spectroscopy

The NMR samples were prepared by dissolving the nucleosides in 9/1 H_2_O/D_2_O. NMR data were acquired on a Bruker Avance II+ instrument operating at 14.1 T. ^1^H NMR spectra were acquired using a double-pulsed field gradient spin-echo (DPFGSE) pulse sequence (27). For **7b** NOESY spectra were acquired at 273, 278, 283 and 288 K with mixing times of 10, 25, 50, 75, 125, 200, 400, 750 and 1000 ms. The size of the data matrices for each spectrum was 2048 × 256 complex data points, the number of scans was 8 and the interscan delay was 1.5 s, yielding a total measuring time of ∼6 h at each temperature.

#### Determination of exchange kinetics

Spectral processing and peak integration were performed using NMRPipe and the NMRDraw software package (28). All subsequent steps were performed using in-house written software written in Matlab (The MathWorks, www.mathworks.com) according to an earlier published protocol (29). Errors in the extracted rate constants were determined by Monte Carlo analysis, where peak intensities were randomly modulated according to the signal to noise levels in the 2D correlation maps.

#### Arrhenius analysis

The temperature dependence of the rate constants for **7b** obtained from the exchange data was obtained by linear regression of ln(kAB) or ln(kBA) versus 1/*T*, respectively, yielding the activation barriers for the *s-cis* to *s-trans* isomerization step.

### Oligodeoxynucleotide synthesis and purification

Starting from preloaded CPG (Proligo), seven 22mer oligodeoxynucleotides (ODNs) for each of the two cytosine modifications, each carrying the respective derivative at a varying position (see Table 1), were custom-synthesized on a 1 μmol scale on an Expedite 8909 DNA/RNA synthesizer (ABI/PerSeptiveBiosystems). A 22mer with the same sequence, however lacking the modifications, was synthesized in a similar fashion (*ODN0*), to serve as reference. After cleavage from the solid support, the DNA strands were purified by means of anion exchange chromatography, eluting with NaClO_4_ in Tris buffer (pH 8). The samples were all gel filtered on NAP^TM^ columns (GE Healthcare) to remove the elution salts. The molecular masses of all synthesized oligonucleotides were confirmed by means of MALDI-TOF mass spectrometry. Examples of these measurements are included in the supporting information section.

### Hybridization and temperature dependent UV-absorption melting experiments

All complementary DNA and RNA strands were obtained from IBA (Goettingen, Germany). Using the in-house synthesized, unmodified DNA (*ODN0*) as reference, the modified ODNs were each hybridized to the complementary DNA strand. The hybridization experiments were carried out in 1× phosphate buffered saline (pH 7.4), with the two complementary strands in a 1:1 ratio, to result in a final duplex concentration of 5 μM. The strands were first incubated at 90 °C for 3 min and duplex formation was allowed at 37 °C over 1 h.

The duplexes were diluted to a final concentration of 0.5 μM in degassed, RNase-free buffer, containing 10 mM NaH_2_PO_4_ (pH 8) and NaCl (45 mM), to a final volume of 800 μL. Melting curves were recorded at 260 nm, a pathlength of 10 mm and with a heating rate of 0.4 °C/min, a slit of 2 nm and a response of 0.2 s. All measurements were carried out at least three times in the temperature ranges between 20 and 85 °C.

To determine the effect of the modification on different helix conformations, the same hybridization and UV-melting experiments were repeated with duplexes formed with complementary RNA strands, also obtained from IBA (Goettingen, Germany). For the oligonucleotides that contain the modifications at positions 10 and 11 from the 5′-end (*ODN10.1*, *ODN11.1*, *ODN10.2* and *ODN11.2* in Table 1) hybridizations were carried out with DNA strands that contain an abasic site directly opposite the modification. As control, the same duplexes were formed with the unmodified DNA strand (*ODN0*).

**Table 1.**
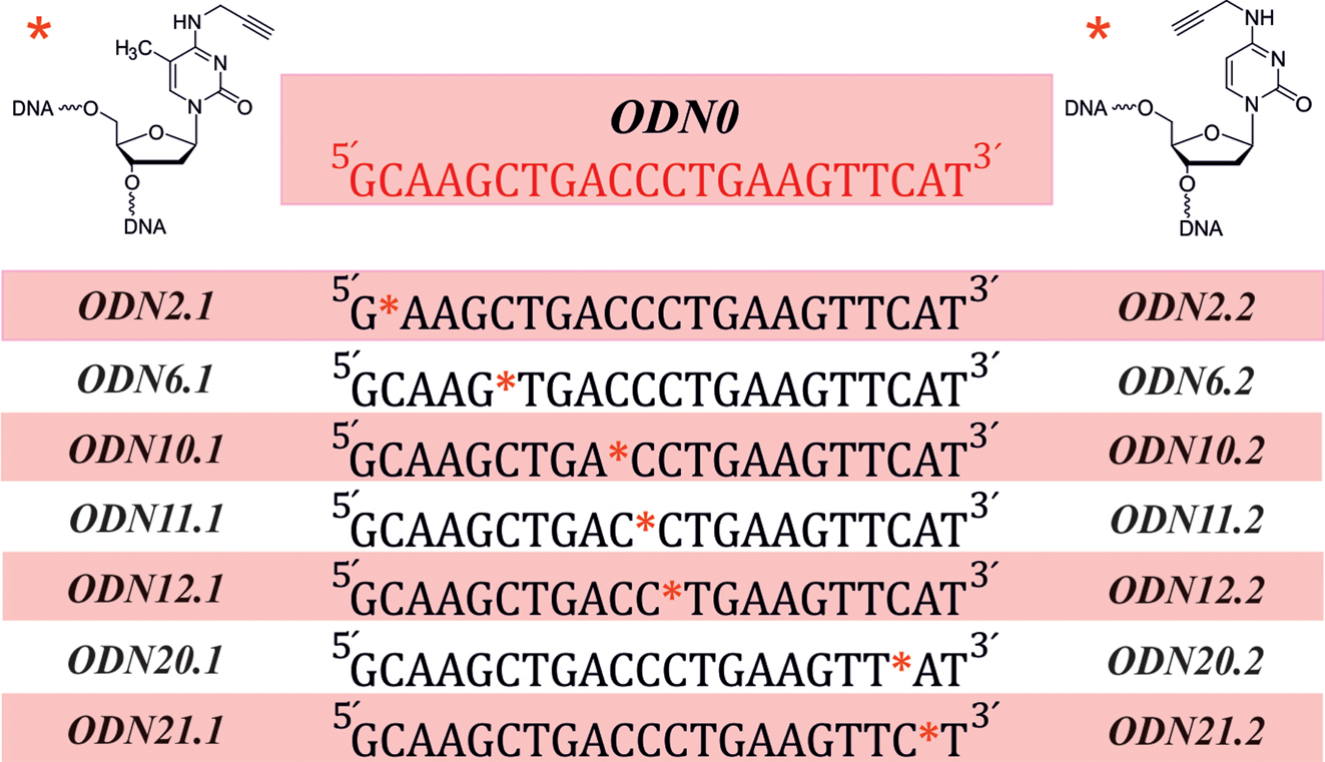
Sequence of the synthesized oligodeoxynucleotides, together with the positions of modifications for each of the two cytosine derivatives.

### Click functionalization

All click reactions were performed in aqueous solutions containing 5% (v/v) DMSO. The solutions were buffered to pH 8 with NaH_2_PO_4_ (100 mM) and contained 10 μM oligonucleotides, 100 μM azido functionalized Atto 647N dye (ATTO-TEC, Siegen, Germany), 0.5 mM CuSO_4_·5H_2_O, 2.5 mM *tris*-[4-(3-hydroxypropyl)-(1, 2, 3)triazolyl-1-methyl]amine (TPTA) and 5 mM sodium ascorbate. The reaction mixtures were agitated under light protection at 25 °C for 2 h.

### Electrophoretic mobility shift assay

Click reactions of single and double strands were analyzed by non-denaturing PAGE. Fifty picomoles of single- and 25 pmol of double-stranded oligonucleotides (based on the measurement of the sense strand) were loaded onto a 15% non-denaturing polyacrylamide gel containing 1× TBE (compounds for nondenaturing PAGE from Carl Roth). PAGE was performed in 1× TBE buffer at temperatures < 30 °C, in order to prevent strand separation. Gels were post-stained for 20 min with Stains-All (Sigma–Aldrich, Munich, Germany) and destained overnight in 75% isopropanol. Detection was carried out on a Typhoon 9400 (GE Healthcare, Munich, Germany), before and after staining, using 633 nm for excitation. Emission signals were recorded at 670 nm.

## RESULTS

To investigate the orientation of a propargyl group on the exocyclic *N4* of cytidines on both the nucleoside level and in an ODN context, we devised a synthetic route to a common precursor, from which both the free nucleoside and the phosphoramidite required for ODN synthesis could each be obtained in a single reaction step.

### Synthesis of modified nucleosides and corresponding phosphoramidites

Starting from deoxyuridine and thymidine, respectively, the key step conversion into cytidines was pursued by a route employing early incorporation of the 5′-*O*-dimethoxytrityl (DMT) group (30), as displayed in Scheme 1. This was followed by a temporary protection of the 3′-OH functionality with *tert*-butyldimethylsilyl (TBDMS), to allow the orthogonal incorporation of an aryl sulfonate ester, i.e. triisopropylbenzenesulfonyl (TPS), onto position four of the nucleobase (30). An interesting observation was the formation of a 2-substituted derivative of up to 15% in relation to the main, 4-substituted product, as determined by ^1^H NMR. This side product was separated from the main product by means of column chromatography, before the subsequent substitution of TPS with propargylamine in an aminolysis reaction. This resulted in the formation of the two respective cytidine derivatives, now containing the key functional group on the exocyclic amine. Deprotection of the 3′-hydroxyl functionality led to **5a/5b**, which yielded the free nucleosides **7a/7b** after cleavage of the 5′-DMT group. Thin layer chromatography of the reaction mixture during detritylation of the two cytosine derivatives indicated the formation of both the tritylated form of thymidine/2′-deoxyuridine (**1a/1b** in supplementary section), as well as their untritylated forms, i.e. thymidine/2′-deoxyuridine. These results suggest a likely deamination of the propargyl residue under acidic conditions, which eventually resulted in a yield of only 70% for both **7a** and **7b**. **5a/5b** were also used to obtain the respective phosphoramidites, **6a/6b** after standard 2′-phosphitylation.

### Orientation of the propargyl moiety on the nucleoside level

**Figure 2.**
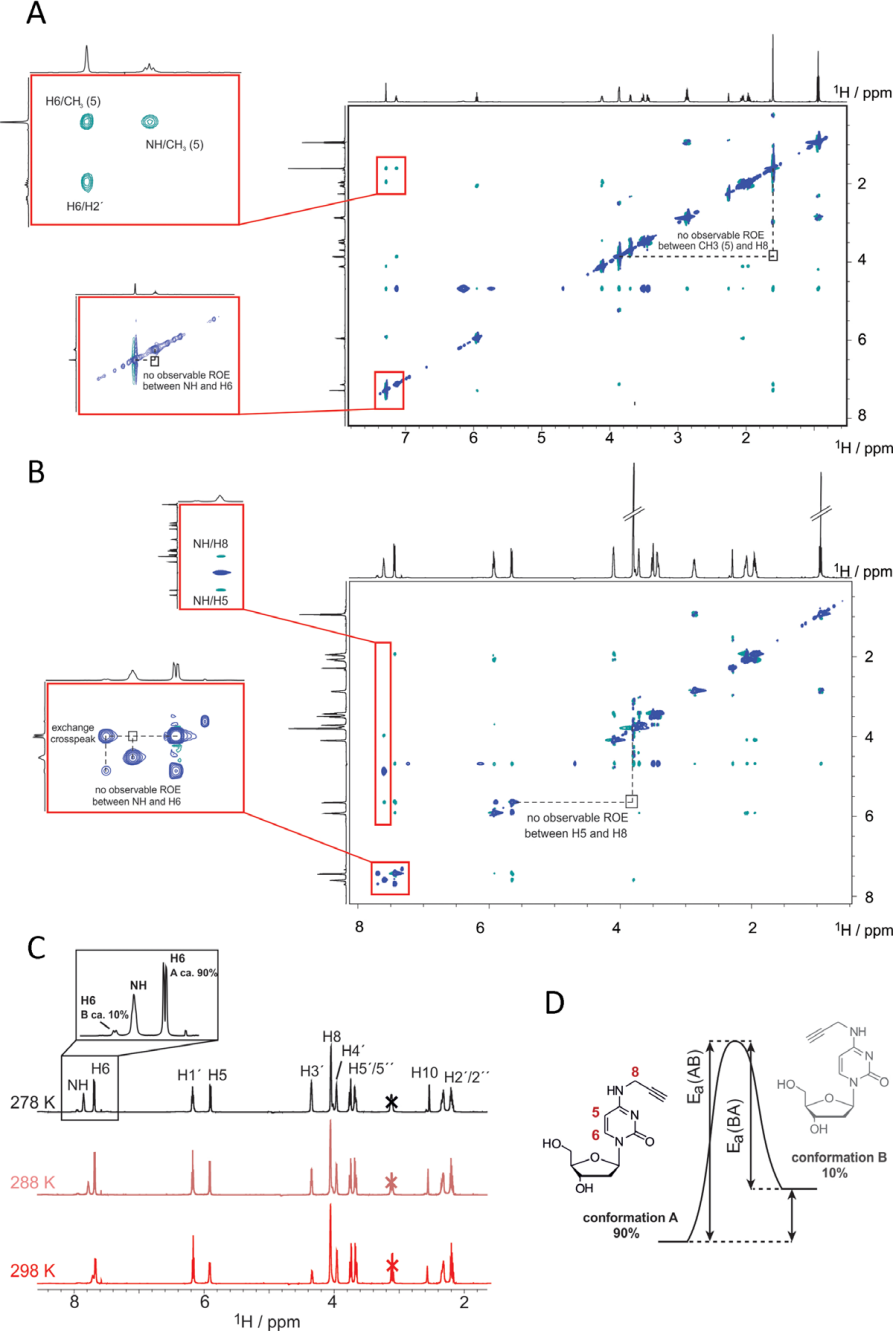
ROESY NMR measurements of the two respective deprotected cytosine derivatives (**7a** and **7b** in Scheme 1), either (**A**) with or (**B**) without a methyl group on position 5. In both cases, the propargyl preferentially adapts an *s-cis*-conformation, but a minor population of *s-trans*-conformation is also found in **7b** by inspection of chemical exchange in the H6 signal (**C**), which was identified in depth at various temperatures to derive activation energy and thermodynamic parameters for the equilibrium between both conformations, as shown in (**D**).

The orientation of the *N^4^*-propargyl moiety on the nucleoside level was evaluated via ROESY NMR in water (H_2_O/D_2_O mixture). The ROESY technique is especially suitable for the detection of spatial proximity between atoms and thus also the degree of rotation around a certain bond via the so-called Nuclear Overhauser Effect (NOE) (24,31). Several potential NOEs could in principle be diagnostic of either an *s-trans* or an *s-cis* conformation, namely between *N^4^*H or H8 and either H5/CH_3_ or H6. While, no NOEs were found for H6 signals in any compound, a CH_3_-*N^4^*H NOE signal in compound **7a** was in evidence (expanded in Figure 2a), while no CH_3_-H8 peak was detectable (highlighted in Figure 2a). Similarly, in compound **7b**, an H5-*N^4^*H peak is in evidence (expanded in Figure 2b), but no H5–H8 signal (highlighted in Figure 2b). These provide strong evidence for *s-cis* being the predominant conformation in both compounds. However, in the ROESY spectrum (Figure 2b) of **7b** acquired at 278 K we found cross peaks for H5 and H6 arising from an exchange process between the *s-cis* and *s-trans* conformation. Based on the analysis of the observed signal pattern of the higher populated (90%) species, conformation A represents the *s-cis* conformation, whereas conformation B (populated to 10%) very likely refers to the *s-trans* conformation. We then addressed the exchange kinetics of the process by acquiring EXSY spectra with varying mixing times, ranging from 10 to 1000 ms at 273, 278, 283 and 288 K (Figure 2c). We could only use the exchange data of H6 for the determination of the exchange kinetics, as resonance overlap impaired the analysis of the kinetic rates using H5. The temperature dependence of the rate constants between conformation A and B for **7b** was used to calculate the activation barriers (Ea(AB) = 27 kcal mol^−1^ and Ea(BA) = 19 kcal mol^−1^) for the *s-cis* to *s-trans* isomerization step, implying a ΔH of 7.81 kcal mol^−1^ between both conformations (Figure 2d, details of the Arrhenius analysis in Supplementary Figure S14 of the supporting information section). Comparison of the relative population of both states at various temperatures placed the difference in Gibbs free energy (Δ*G*) around 1.1–1.4 kcal mol^−1^.

These findings are in agreement with above mentioned investigations on *N^4^*-methylcytidine on the nucleotide level (24,25), which report a preference for the *cis*-conformation as well. Given the numerous reports on *N^4^*-methylcytidine in helical structures of oligonucleotides (10–12,20–23), we hypothesized that *N^4^*-propargylcytidine might behave similarly and, in the structural context of a Watson–Crick helix, might alter the orientation of the propargyl group, directing it away from the Watson–Crick face. The free energy required would presumably stem from hybridization. We have therefore set out to test this hypothesis.

#### The propargyl group within a double helix

To verify the above mentioned hypothesis in an oligonucleotide context, both cytidine derivatives were incorporated into DNA strands. An unmodified oligodeoxynucleotide (*ODN0*) as shown in Table 1 was synthesized as reference. Modified oligonucleotides contained propargylated cytidines replacing individual single cytidines within the DNA sequence. The nomenclature applied here uses the position of substitution counted from the 5′, and the type of modification, where 1 corresponds to the 5-methyl-*N^4^-*propargylaminyl-2′-deoxycytidine and 2 corresponds to *N^4^-*propargylaminyl-2′-deoxycytidine (Table 1).

The DNA strand corresponds in sequence to the passenger strand of an anti-eGFP siRNA which we had used in previous studies (16). To investigate the stability of helices containing propargylated cytidines, the respective oligodeoxynucleotides were thus hybridized (i) to an oligoribonucleotide identical to the antisense strand of the corresponding siRNA, and (ii) to an oligodeoxynucleotide of identical sequence. Determination of melting temperature would thus inform on perturbed stability in (i) the A-type helices of RNA-DNA hybrids and (ii) B-type DNA-DNA helices, respectively. Of note, the resulting duplexes are of classical siRNA design with a two-nucleotide overhang on each 3′-end, such that the substitution of A21 against a modified cytidine cannot be expected to disrupt a base pair.

Results of UV melting curves, as depicted in Table 2, show relatively minor variations (maximum decrease of 3 °C) between the unmodified duplexes and duplexes of all oligodeoxynucleotides of the ODN*x*.2 series, i.e. those containing propargylated cytidines, but no methyl-C5. However, where the introduction of a single methyl-C5 has been reported to increase melting temperatures of DNA helices (32), we find that the *double* modification of propargyl-*N^4^* and methyl-C5 results in a pronounced and position-specific negative effect on duplex stability (ODN*x*.1 series). While modifications at either extremity show no or weak effects, helical destabilization is extremely pronounced in the center of the duplex, with the strongest decrease of 11 °C observed in a DNA–RNA hybrid of ODN12.1 with presumed A-form helix.

**Table 2.**
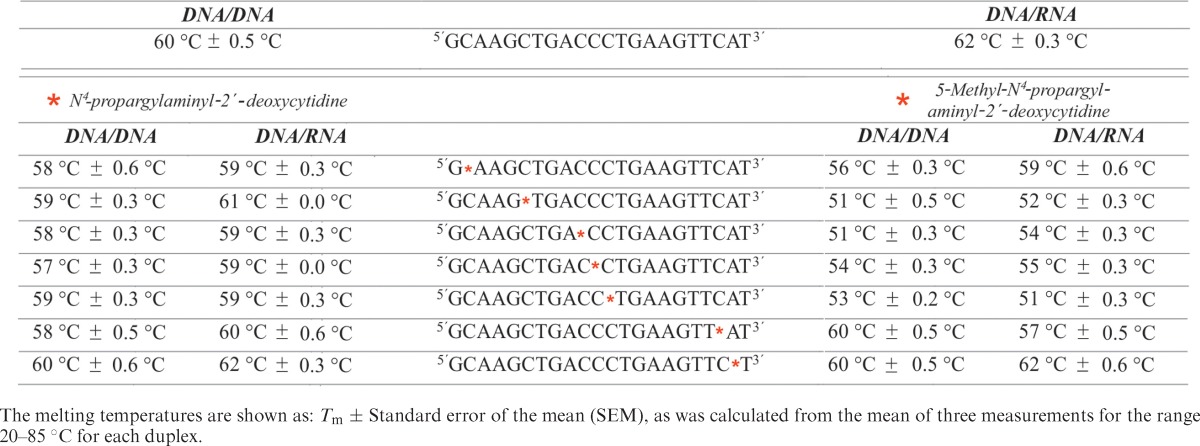
Melting temperatures obtained for duplexes formed between the synthesized DNA strands and complementary DNA and RNA strands, respectively, where * indicates the position of modification.

**Figure 3.**
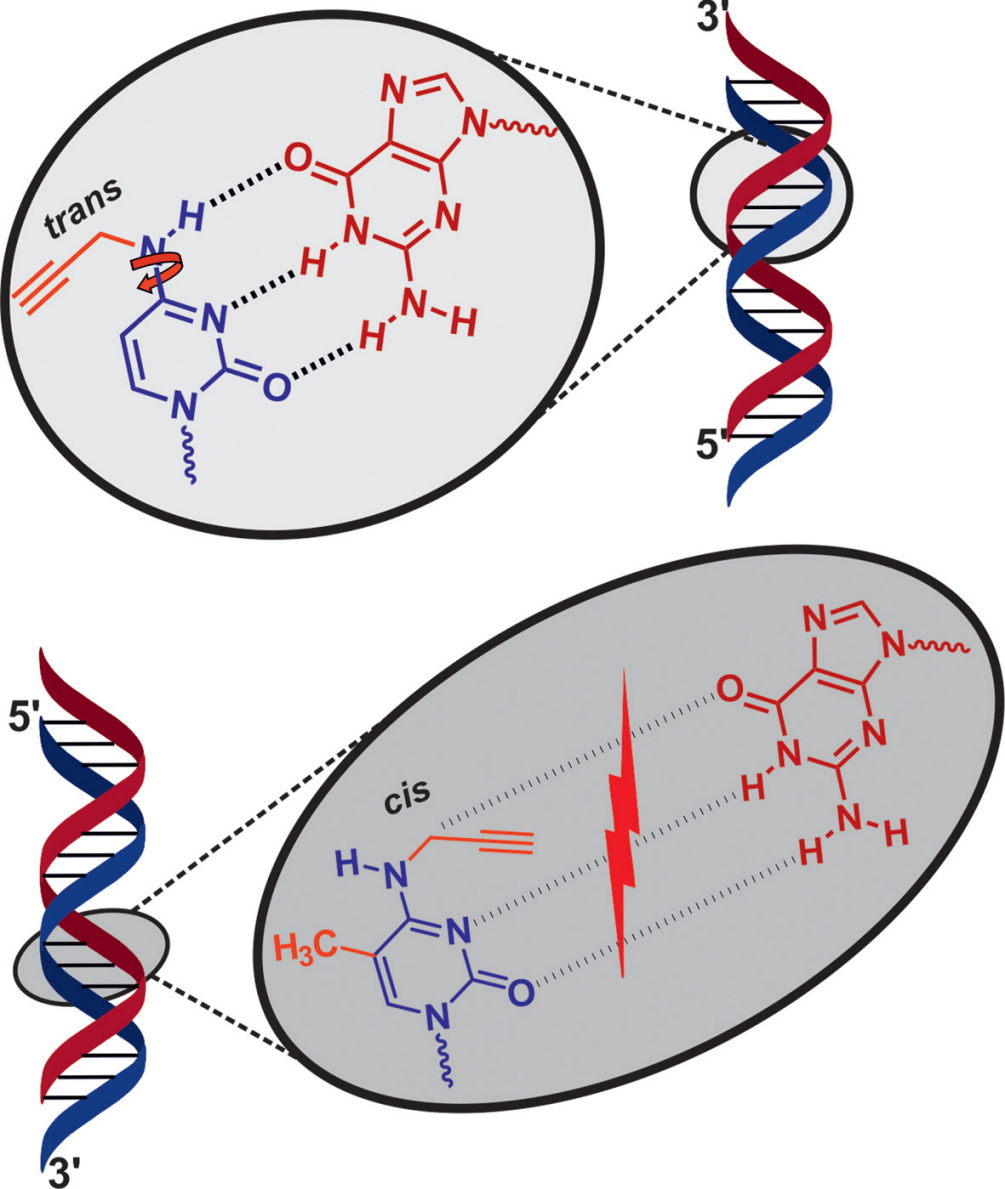
Proposed Watson–Crick base pairing for *N^4^*-substituted cytidine derivatives both in the absence and presence of a methyl group on position 5. The 5-CH_3_ likely prevents rotation of the propargyl group away from the Watson–Crick edge during base pairing, thus abolishing such H-bonding completely.

While the results of the ODN*x*.2 series are compatible with a minor energy penalty paid for the propargyl group rotating away from the Watson–Crick face to allow standard base pairing, the drastic decrease in the center of the ODN*x*.1 series implies major interference with standard Watson–Crick interactions. Presumably, the rotation of the propargyl group away from the Watson–Crick face and toward the Hoogsteen edge/‘CH’-edge is prevented by the bulk of the methyl group on C5, and the propargyl group is still pointed to sterically interfere with Watson–Crick pairing, as depicted in Figure 3.

**Figure 4.**
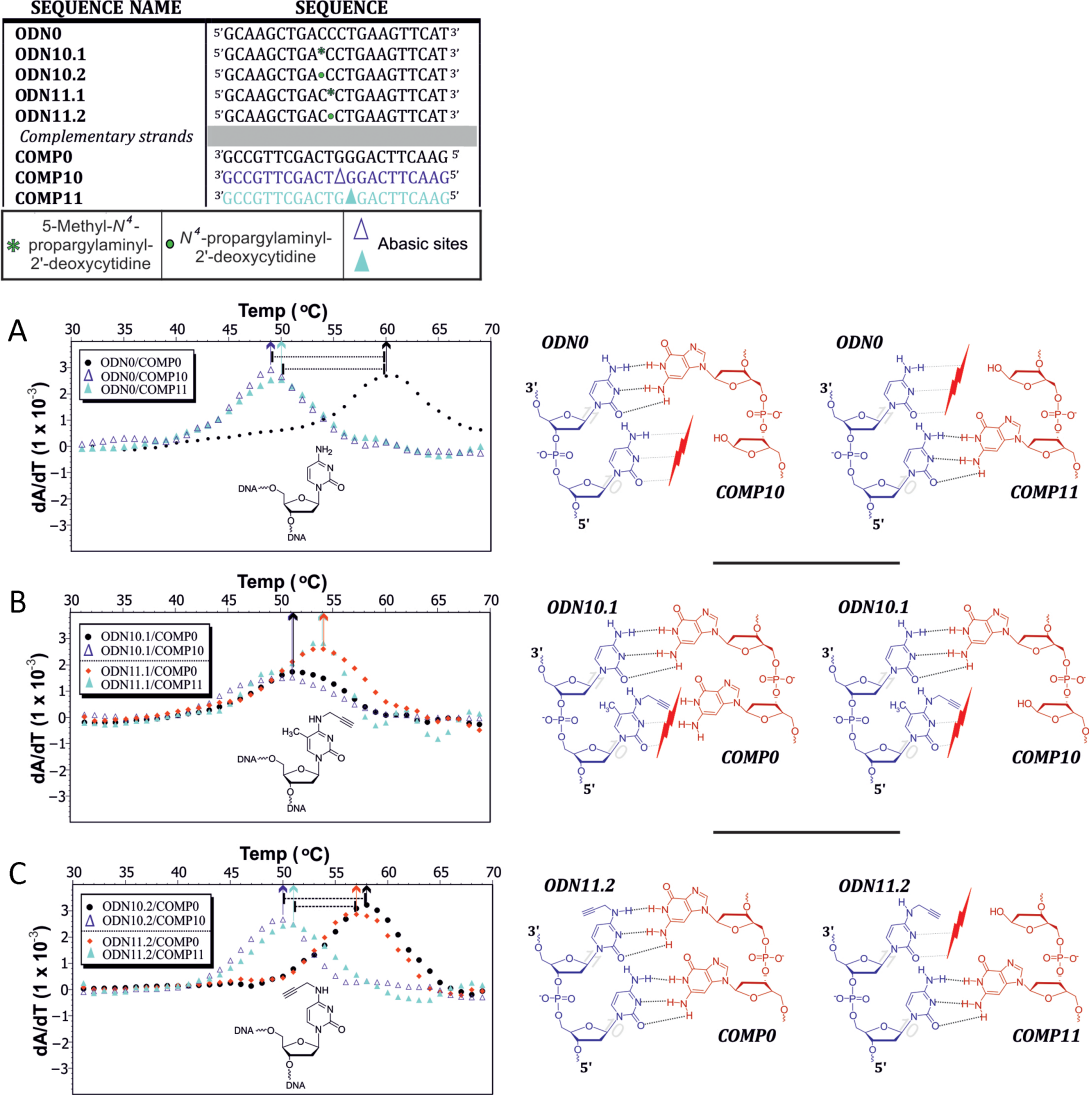
Comparison of melting temperatures obtained between duplexes both with and without abasic sites, presented as first derivatives of temperature dependent UV-absorption melting curves for duplexes formed between (**A**) the unmodified strand and two complementary strands, each carrying an abasic site at a different internal position (dark and light blue), as well as one complementary strand lacking an abasic site (black). Similar duplexes were formed with the modified strands: (**B**) m^5^C-derivative and (**C**) cytidine derivative lacking the methyl group on position 5.

To verify this hypothesis, we recorded melting curves of duplexes of selected oligonucleotides of the ODNx.1 series, with DNA strands featuring abasic sites opposite the selected modified positions: ODN10.1 (or 10.2, respectively) was hybridized to *COMP10* and ODN11.1/11.2 were hybridized to *COMP11* (Figure 4). These correspond to duplexes lacking the opposing guanosine, which leaves no Watson–Crick partner, but provides space to alleviate a steric clash caused by the propargyl group. Melting curves are shown in Supplementary Figure S16 (supporting information), and derivatives of the melting curves are given in Figure 4. Figure 4a compares melting of duplexes of unmodified *ODN0* and either of the two abasic ODNs to the reference DNA duplex, clearly showing that a Watson–Crick gap in the middle of the helix destabilizes by ∼10 °C in melting temperature for the 10.x position, and by 6 °C for the 11.x position. Thus, oligodeoxynucleotides containing the singly modified cytidine derivative, i.e. ODNs*x*.2, form duplexes with comparable stability to normal duplexes. However, for the doubly modified cytidine oligodeoxynucleotides, melting behavior mimicked that of duplexes that have no base pair at either position 10 or 11. This underscores the suspicion that the methyl containing cytidine derivatives cannot enter into Watson–Crick base pairs. Of note, numerous stable alternative base pairs have been described in A- and B-form helices, which may employ the Hoogsteen edge (33) or which do not rely on hydrogen bonds, but exclusively on the stabilizing contribution of base stacking (34,35). Our melting data only give occasional suggestions of the impact of base stacking in variegated sequence context. For example, the melting temperatures of various constructs change depending on the position of the modified cytidine (compare duplexes containing ODN10.2 versus ODN11.2 and ODN10.1 versus 10.2 in Figure 4b, c). However, our understanding of these contexts is not advanced enough for a more detailed interpretation.

In summary, the melting data lead to the conclusion that the monosubstituted *N^4^*-propargyl cytidines integrates well into a helix with melting temperatures only slightly below unmodified duplexes. In contrast, an additional CH_3_-substituent, away from the Watson–Crick face on the ‘CH’-edge, leads to drastic and position-specific drops in helix stability, which is comparable to duplexes in which base pairing at the respective site is ablated by introduction of an abasic site.

**Figure 5.**
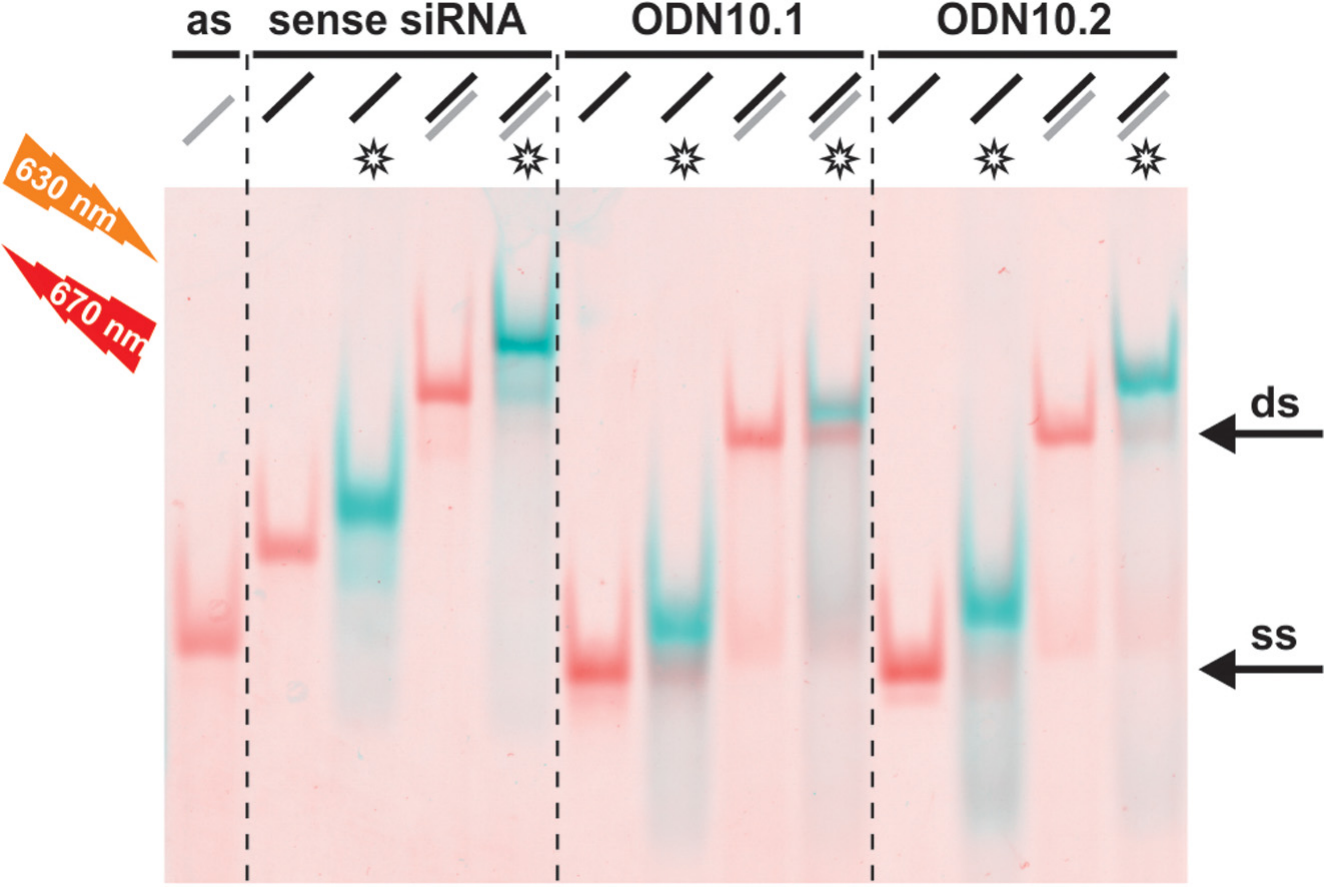
Comparison of the click efficiency of the single stranded (ss), alkyne modified oligonucleotides MH662 (commercial, left), ODN10.1, ODN10.2, as compared to their double strands (ds), formed with an antisense siRNA (as). Atto647N azide was used as azido functionalized dye. For analysis of click efficiency, native polyacrylamide gel electrophoresis was followed by fluorescence scanning at different wavelengths to distinguish unclicked and clicked (*) oligonucleotides (see blue bands). Excitation, before (blue) and after staining (red) with Stains-All, was done at 633 nm. Emission signals were recorded at 670 nm.

In order to test the ‘click’-ability of the propargyl-containing oligodeoxynucleotides, we applied ODN10.1 and ODN10.2, and their double strands (ds) with an antisense siRNA strand (as) respectively, in a Cu(I)-catalyzed-azide-alkyne-[3+2]-cycloaddition (CuAAC) reaction with the fluorescent dye Atto 647N azide. After removal of residual dye, the nucleic acids were separated by non-denaturing PAGE, which allowed to distinguish between double and single stranded DNA. Also a significant shift in mobility of the clicked species allowed an assessment of the click efficiency. Fluorescence of the attached dyes was imaged before staining with Stains-All and renewed imaging, which then identified the unlabeled species as well. The results, which are depicted as an overlay in Figure 5, show that the cytidines are only marginally less efficiently labeled than a commercial oligonucleotide containing a hexynyl group as ‘clickable’ moiety. Despite presumed structural differences of the duplexes, labeling efficiency was not significantly affected by hybridization. Further experiments under milder conditions (not shown), resulted in lower labeling yields, but still did not reveal any significant differences between the differentially modified cytosines (**7a** or **7b**) that might be interpreted in terms of reduced accessibility of the alkynyl group in any of the nucleic acids.

## DISCUSSION

A single substituent on the *N4* of cytosine has the possibility to adopt either a *cis-* or a *trans-*conformation, depending on surrounding influences. According to previous studies of methyl groups as substituents, the limited rotation around the *C^4^–N^4^* bond in cytosine likely leads to a *cis*-coordinated structure (24), which, in our case, would direct the *N^4^*-functionality toward the Watson–Crick edge, at least on the nucleoside level. Our NOESY experiments confirm this for a propargyl substituent on the nucleoside level.

It has been speculated (25) that in the context of an ODN, hybridization to a complementary strand might provide the free energy needed to rotate the substituent to the ‘CH’-edge to allow Watson–Crick base pairing. We find that for both B-form helices (DNA/DNA) and A-form helices (DNA/RNA), this small penalty is reflected in melting temperatures lowered by as little as one or two degrees throughout all positions within the helix (Table 2, left two columns). The melting data lead to the conclusion that the monosubstituted *N^4^*-propargyl cytidines integrate well into a helix, which implies rotating the substituent to the ‘CH’-edge to allow base pairing. In contrast, the same mechanism is not possible when the ‘CH’-edge is blocked by a 5-methyl group, consequently leading to disruption of Watson–Crick base pairing, with a destabilizing effect on helices that is most pronounced at center positions of the helix. Figuratively spoken, the propargyl group is torqued into a position where it interferes with standard base pairing by the methyl group.

In conclusion, we can confirm the idea that a single alkylation of the exocyclic amine of nucleosides may be tolerated during duplex formation (17–20), and extend this finding to the propargyl residue on the *N4* of cytidines. For the purpose of further nucleic acid functionalization, the terminal alkyne of our propargyl functionality remains a handy handle for click applications. Such applications may involve CuAAC-mediated labeling after transfection of oligonucleotides into cells to investigate subcellular distribution, as has been exemplifed on the nucleoside level by Jao and Salic, using ethynyluridine (36). Further studies may involve structural and biological effects of triazole linkages as formed upon CuAAC as, for example, published by Beal *et al*. (37).

## SUPPLEMENTARY DATA

Supplementary Data are available at NAR Online.

SUPPLEMENTARY DATA
