# Supplementary material for: Intermolecular ‘cross-torque’: the N4-cytosine propargyl residue is rotated to the ‘CH’-edge as a result of Watson–Crick interaction
Source: Nucleic Acids Res. 2015 Apr 30;43(11):5275–83. doi: 10.1093/nar/gkv285 (PMC4477647; doi:10.1093/nar/gkv285)
Supplement: SUPPLEMENTARY DATA [file supp_43_11_5275__index.html]

Intermolecular ‘cross-torque’: the N4-cytosine propargyl residue is rotated to the ‘CH’-edge as a result of Watson–Crick interaction — SUPPLEMENTARY DATA 

# Intermolecular ‘cross-torque’: the *N4*-cytosine propargyl residue is rotated to the ‘CH’-edge as a result of Watson–Crick interaction

## SUPPLEMENTARY DATA

**Files in this Data Supplement:**

- SUPPLEMENTARY DATA
