# Supplementary material for: Intermolecular ‘cross-torque’: the N4-cytosine propargyl residue is rotated to the ‘CH’-edge as a result of Watson–Crick interaction
Source: Nucleic Acids Res. 2015 Apr 30;43(11):5275–83. doi: 10.1093/nar/gkv285 (PMC4477647; doi:10.1093/nar/gkv285)
Supplement: SUPPLEMENTARY DATA [file supp_gkv285_Supplementary_Data_NAR-03207-F-2014.R2_Revision_New.pdf]

Supplementary Data

Intermolecular "cross-torque": the *N*[4]-cytosine propargyl group is rotated to the "CH"-edge as a result of Watson-Crick interaction

Olwen Domingo[1], Isabell Hellmuth[1], Andres Jäschke[2], Christoph Kreutz[3] and Mark Helm[1]*

[1]Department of Pharmaceutical Chemistry, Institute of Pharmacy and Biochemistry, Johannes Gutenberg University, Mainz, Rheinland-Palatinate, 55128, Germany

[2]Institute of Pharmacy and Molecular Biotechnology, Heidelberg University, Heidelberg, Baden-Wuerttemberg, 69120, Germany

[3]Institute of Organic Chemistry, University of Innsbruck, Innsbruck, Tyrol, 6020, Austria

* To whom correspondence should be addressed. Tel: +49 6131 39 25731; Fax: +49 6131 39 20373; Email: mhelm@uni-mainz.de

TABLE OF CONTENTS

PAGE

Syntheses of 2´-Deoxycytidine Derivatives

General Experimental 3

Synthesis of 3´-*O*-CEP-5´-*O*-DMT-5-methyl-*N*^{4}-propargyl-2´-deoxycytidine (**6a**) 5
Synthesis of 5-Methyl-*N*^{4}-propargyl-2´-deoxycytidine (**7a**) 11
Synthesis of 3´-*O*-CEP-5´-*O*-DMT-*N*^{4}-propargyl-2´-deoxycytidine (**6b**) 12
Synthesis of *N*^{4}-Propargyl-2´-deoxycytidine (**7b**) 18

Spectra of 3´-*O*-CEP-5´-*O*-DMT-5-methyl-*N*^{4}-propargyl-2´-deoxycytidine (**6a**)

Figure S1. ^{1}H NMR 19
Figure S2. ^{13}C NMR 19
Figure S3. ^{31}P NMR 20
Figure S4. ESI-MS 20

Spectra of 5-Methyl-*N*^{4}-propargyl-2´-deoxycytidine (**7a**)

Figure S5. ^{1}H NMR 21
Figure S6. ESI-MS 21
Figure S7. Temperature dependent ^{1}H NMR spectra 22

Spectra of 3´-*O*-CEP-5´-*O*-DMT-*N*^{4}-propargyl-2´-deoxycytidine (**6b**)

Figure S8. ^{1}H NMR 23
Figure S9. ^{13}C NMR 23
Figure S10. ^{31}P NMR 24
Figure S11. ESI-MS 24

Spectra of *N*^{4}-Propargyl-2´-deoxycytidine (**7b**)

Figure S12. ^{1}H NMR 25
Figure S13. ESI-MS 25
Figure S14. Temperature dependent ROESY NMR experiments and Arrhenius analysis of (**7b**) 26

Oligonucleotide Synthesis and Work-up Procedure 27

Table S1. Sequences of synthesised oligodeoxynucleotides 27
Figure S15. MALDI-TOF spectra of selected in-house synthesised oligomers 28
Figure S16. Temperature dependent UV-absorption melting curves 29

Syntheses of 2´-Deoxycytidine Derivatives

General Experimental

Chemical reagents and solvents were purchased from various commercial suppliers. For the purpose of manual liquid chromatography procedures, organic solvents were distilled prior to use. Deuterated solvents were purchased from Deutero GmbH (Kastellaun, Germany).

Thin layer chromatography (**TLC**): Pre-coated silica gel plates, Polygram® Sil G/UV_{254} (40 x 80 mm) from Macherey-Nagel (Dueren, Germany) were used for monitoring chemical reactions. Compound spots were visualised with UV-light at λ = 254 nm and/or Seebach's reagent, consisting of phosphor-molybdic acid (2.50 g), $Ce(SO_4)_2$ (1.00 g) and diluted H_2SO_4 (94–96% H_2SO_4: 6 mL, water: 94 mL). Orange-coloured spots are formed upon treatment of the DMT containing compounds with the reagent. The spots turn blue in almost all cases by heating them to about 90 °C.

Column chromatography (**CC**): Silica gel 60 (230-400 mesh) was purchased from Sigma-Aldrich (Taufkirchen, Germany; product manufactured by Fluka, Buchs, Switzerland) and Merck KGaA (Darmstadt, Germany), respectively. Basic aluminium oxide 90 (0.063-0.200 mm, activity stage I) was purchased from Merck KGaA.

Mass spectrometry: Mass spectra were obtained from various instruments depending on the required technology: ESI spectra: Micromass LCT spectrometer; HR-ESI spectra: Bruker MicroTOF-Q II spectrometer; FD spectra: Finnigan MAT 95 spectrometer; MALDI-TOF spectra: Bruker BIFLEX III.

IR spectroscopy: IR spectra were recorded with a Nicolet Avatar 330 FT-IR spectrometer (Thermo Electron Corporation, USA).

NMR-spectroscopy: NMR spectra were obtained from various instruments: Bruker AC 300 MHz (^{1}H: 300 MHz, ^{13}C: 75 MHz), Bruker 400 MHz (^{1}H: 400 MHz, ^{13}C: 100 MHz) and Bruker Avance III HD 400 MHz (^{31}P: 162 MHz), respectively.

^{1}H and ^{13}C NMR spectra were standardised using solvent resonances: DMSO-d_6: δ_H = 2.500, δ_C = 39.50 ppm. ^{31}P NMR spectra were standardised externally with 85% phosphoric acid: δ_P = 0.000 ppm. All ^{13}C and ^{31}P spectra were proton decoupled. Chemical shifts (δ) are in ppm and the multiplicity of the ^{1}H NMR resonances is abbreviated in the following way: d = doublet, m = multiplet, q = quartet, sept = septet, s = singlet, t = triplet. The stars (*) inside the figures of the NMR spectra denote signals of impurities present in the substances.

Numbering of atoms

For the purpose of the assignment of signals obtained from the NMR spectra of the synthesised compounds, i. e. carbon atoms, CH and OH protons, respectively, the following numbering of the 2´-deoxynucleoside scaffold as well as the propargyl group is used:

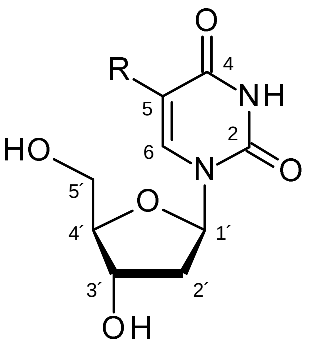

R = H, CH_3

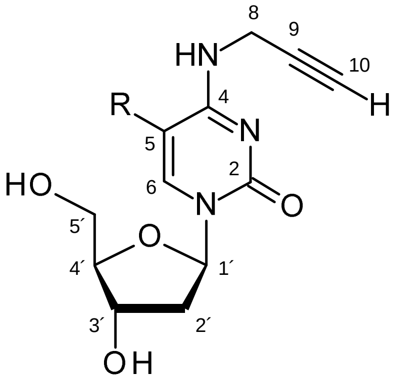

Abbreviations

Abbreviations used for chemicals and chemical moieties:

CEP-Cl	= 2-Cyanoethyl-*N*,*N*-diisopropylphosphoramido chloridite
CEP	= 2-Cyanoethyl-*N*,*N*-diisopropylphosphoramido
DIPEA	= Diisopropylethylamine (*Hünig´s base*)
DMAP	= 4-Dimethylamino pyridine
DMT-Cl	= 4,4´-Dimethoxytriphenylmethyl chloride
DMT	= 4,4´-Dimethoxytriphenylmethyl
TBAF	= Tetrabutylammonium fluoride
TBDMS-Cl	= *tert*-Butyldimethylsilyl chloride
TBDMS	= *tert*-Butyldimethylsilyl
TFA	= Trifluoroacetic acid
TPS-Cl	= 2,4,6-Triisopropylbenzenesulfonyl chloride
TPS	= 2,4,6-Triisopropylbenzenesulfonyl

Abbreviations of chemical moieties are used as part of the chemical names of the synthesised compounds as well as for assignments of their NMR signals.

Synthesis of 3´-*O*-CEP-5´-*O*-DMT-5-methyl-*N*[4]-propargyl-2´-deoxycytidine (**6a**)

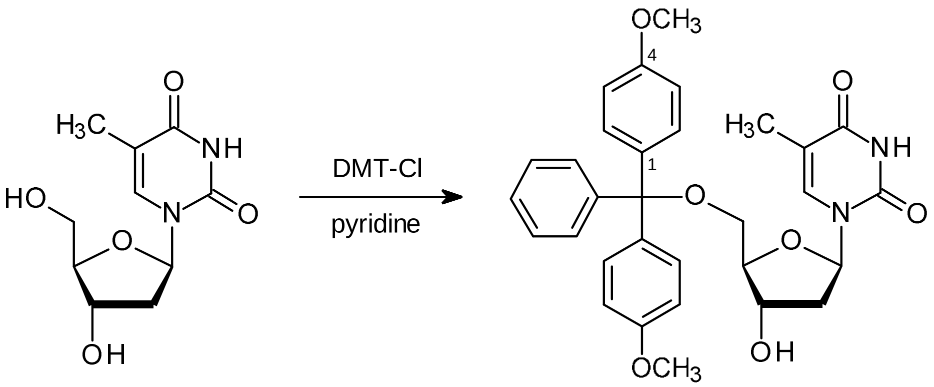

1a

5´-*O*-DMT-thymidine (**1a**). Under Ar, to a solution of thymidine (610 mg, 2.50 mmol) in dry pyridine (10 mL) a solution of DMAP (153 mg, 1.25 mmol, 0.5 equiv) in dry pyridine (2 mL) was added, followed by slow and dropwise addition of DMT-Cl (1.3 g, 3.00 mmol, 1.2 equiv) in a minimal amount of dry pyridine. The mixture was stirred at ambient temperature under an argon atmosphere and the reaction was monitored by TLC. After complete consumption of the starting material (12 hours), MeOH (2 mL) was added and the resulting mixture was concentrated under vacuum. Purification of the residue by CC (silica gel) with DCM/MeOH (15:1 → 10:1, containing 1% triethylamine) afforded a colourless foam (1.28 g, 2.35 mmol, 94%, m.p. 225–228 °C). TLC: silica gel, DCM/MeOH (15:1), R_f = 0.43. ^{1}H NMR (400 MHz, DMSO-d_6) δ = 1.44 (3H, d, ^4J = 0.9 Hz, 5-CH_3), 2.20 (ABXY, 2H, m, H-2´$_{A,B}$), 3.18 (ABX, 2H, m, $^2J_{AB}$ = 10.5 Hz, H-5´$_{A,B}$), 3.73 (6H, s, 2 x OCH_3), 3.88 (ABX, 1H, m, H-4´$_X$), 4.32 (ABXY, 1H, m, H-3´$_Y$), 5.36 (1H, d, ^3J = 4.4 Hz, 3´-OH), 6.20 (ABXY, 1H, m, H-1´$_X$); DMT: aryl (AA´BB´, ^3J = 8.9 Hz): 6.89 (4H, $H_{A,A´}$), 7.25 (4H, $H_{B,B´}$), phenyl (AA´BB´C, ^3J = 7.3 Hz, H_C overlapped by $H_{B,B´}$ aryl): 7.31 (2H, $H_{B,B´}$), 7.38 (2H, $H_{A,A´}$); 7.51 (1H, q, ^4J = 0.9 Hz, H-6), 11.35 (1H, s, NH).
^{13}C NMR (100 MHz, DMSO-d_6), thymine moiety: δ = 11.73 (5-CH_3), 109.59 (C-5), 135.71 (C-6), 150.39 (C-2), 163.68 (C-4), 2´-deoxyribose moiety: 39.5 (C-2´, hidden under the solvent), 63.79 (C-5´), 70.55 (C-3´), 83.75 (C-1´), 85.49 (C-4´); DMT: 55.06 (OCH_3), 85.84 (quat. C, DMT, aliphatic carbon), 113.25 (*o*-CH, aryl), phenyl (CH): 126.79, 127.67, 127.92; 129.75 (*m*-CH, aryl), *ipso*-C (phenyl): 135.26, 135.45; *ipso*-C (aryl): 144.73, 158.16. FT-IR (neat): $\tilde{\nu}$ = 2876, 1683, 1609, 1380, 1356, 1249, 1033 (cm^{-1}). FD-MS: *m/z* $[M]^+$ calcd for $C_{31}H_{32}N_2O_7$: 544.2; found: 544.7.

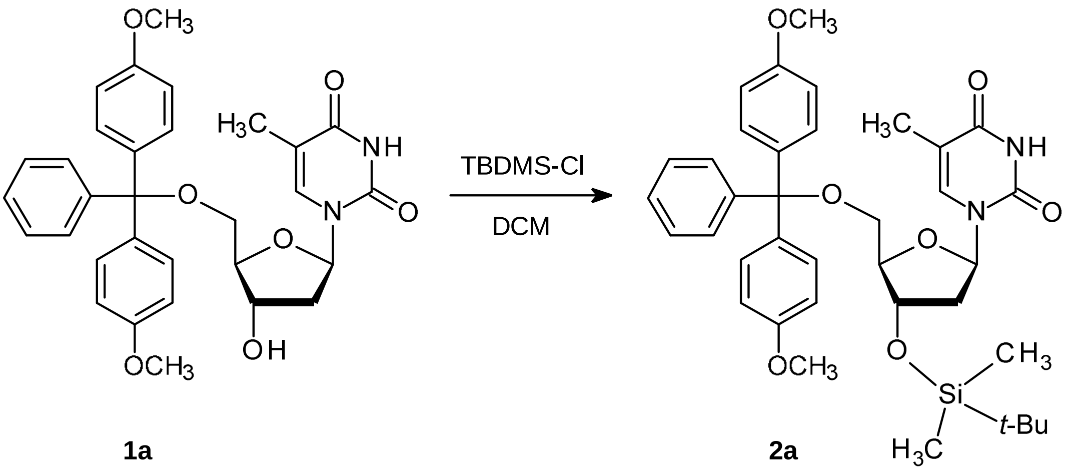

1a **2a**

3´-*O*-TBDMS-5´-*O*-DMT-thymidine (**2a**). Under Ar, **1a** (3.50 g, 6.43 mmol) was dissolved in dry DCM (10 mL), followed by the consecutive addition of imidazole (1.90 g, 28.20 mmol, 4.4 equiv) and TBDMS-Cl (4.20 g, 27.87 mmol, 4.0 equiv), both as a solution in a minimal amount of dry DCM. The mixture was stirred at ambient temperature until TLC indicated complete consumption of the starting material (12 hours). MeOH (2 mL) was added and the solution was poured into EtOAc (70 mL). The organic layer was extracted with 5% aqueous $NaHCO_3$ (2 x 50 mL) and washed with water (1 x 50 mL) and brine (1 x 50 mL). Drying of the organic layer with anhydrous $MgSO_4$ and evaporation to dryness gave a colourless solid (3.64 g, 5.53 mmol, 86%, m.p. 108–112 °C). TLC: silica gel, DCM/MeOH (20:1), R_f = 0.81. ^{1}H NMR (400 MHz, DMSO-d_6) δ = –0.05 (3H, s, Si-CH_3), 0.00 (3H, s, Si-CH_3), 0.78 (9H, s, Si-*t*-Bu), 1.51 (3H, m, 5-CH_3), 2.22 (ABXY, 2H, m, H-$2´_{A,B}$), 3.20 (ABX, 2H, m, $^2J_{AB}$ = 10.5 Hz, H-$5´_{A,B}$), 3.73 (6H, s, 2 x OCH_3), 3.81 (ABX, 1H, m, H-$4´_X$), 4.45 (ABXY, 1H, m, H-$3´_Y$), 6.15 (ABXY, 1H, m, H-$1´_X$); DMT: aryl (AA´BB´, ^3J = 11.2 Hz): 6.89 (4H, $H_{A,A´}$), 7.25 (4H, $H_{B,B´}$), phenyl (AA´BB´C, ^3J = 7.5 Hz, H_C overlapped by $H_{B,B´}$ aryl): 7.31 (2H, $H_{B,B´}$), 7.39 (2H, $H_{A,A´}$); 7.54 (1H, m, H-6), 11.36 (1H, s, NH). ^{13}C NMR (100 MHz, DMSO-d_6), thymine moiety: δ = 11.86 (5-CH_3), 109.56 (C-5), 135.83 (C-6), 150.31 (C-2), 163.68 (C-4); 2´-deoxyribose moiety: 39.69 (C-2´, partially overlapped by solvent), 62.94 (C-5´), 71.51 (C-3´), 83.75 (C-1´), 85.17 (C-4´); DMT: 55.02 (OCH_3), 85.93 (quat. C, DMT, aliphatic carbon), 113.19 (*o*-CH, aryl), CH (phenyl): 126.80, 127.64, 127.86; 129.69 (*m*-CH, aryl), *ipso*-C (phenyl): 135.21, 135.32; *ipso*-C (aryl): 144.59, 158.19; TPDMS: –5.14, –4.82 (Si-CH_3); 17.59 (quat. C, Si-*t*-Bu), 25.58 (CH_3, Si-*t*-Bu). FT-IR (neat): $\tilde{\nu}$ = 2945, 1687, 1605, 1397, 1380, 1364, 1249, 1094, 829 (cm^{-1}). FD-MS: *m/z* $[M]^+$ calcd for $C_{37}H_{46}N_2O_7Si$: 658.3; found: 658.6.

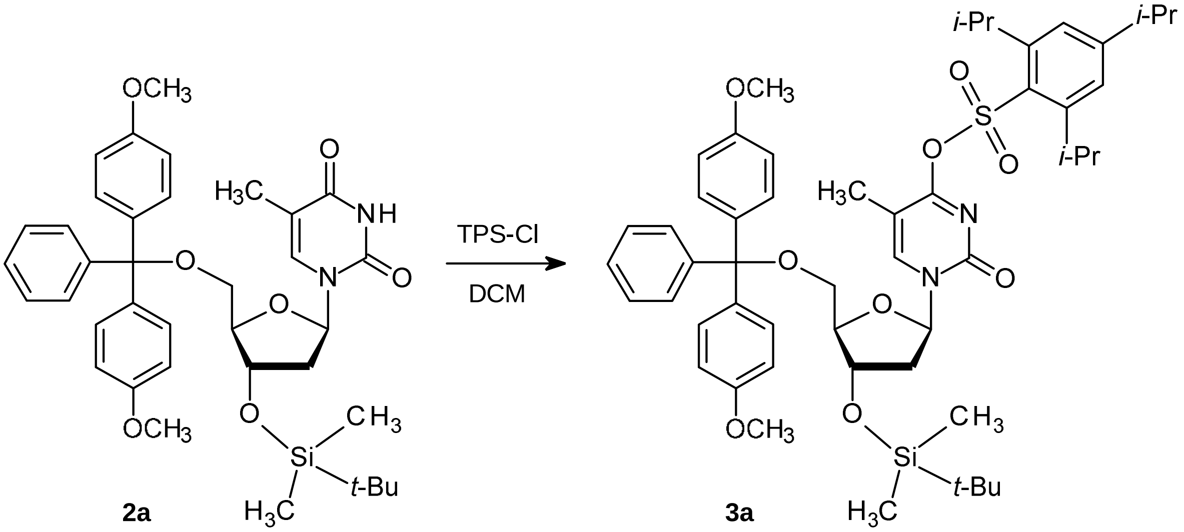

3´-*O*-TBDMS-5´-*O*-DMT-*O*[4]-TPS-thymidine (**3a**). Under Ar, to a stirred solution of **2a** (1.30 g, 1.97 mmol) in dry DCM (10 mL), DMAP (29.00 mg, 0.18 mmol, 0.09 equiv) and triethylamine (1.30 mL, 9.28 mmol, 4.70 equiv) were added. Subsequently, a solution of TPS-Cl (0.72 g, 2.36 mmol, 1.20 equiv) in a minimal amount of dry DCM was added dropwise and the solution was stirred at ambient temperature overnight until TLC analysis showed approximately 60% (estimation based on spot sizes) conversion of the starting material to product. Concentration of the reaction mixture under vacuum and purification of the remaining pale yellow oil by CC (aluminium oxide, basic) with cyclohexane/EtOAc (3:1) yielded a colourless solid (0.88 g, 0.95 mmol, 50%, m.p. 108–111 °C). TLC: silica gel, cyclohexane/EtOAc (3:1), R_f = 0.70. ^{1}H NMR (400 MHz, DMSO-d_6) δ = –0.10 (3H, s, Si-CH_3), –0.03 (3H, s, Si-CH_3), 0.74 (9H, s, Si-*t*-Bu), 1.21 (18H, m, CH_3, *i*-Pr), 1.61 (3H, m, 5-CH_3), 2.26 (ABXY, 2H, m, H-$2´_{A,B}$), 2.96 (1H, m, CH, *p*-*i*-Pr), 3.26 (ABX, 2H, m, H-$5´_{A,B}$, partially hidden under H_2O), 3.73 (6H, s, 2 x OCH_3), 3.88 (ABX, 1H, m, H-$4´_X$), 4.18 (2H, m, CH, *o*-*i*-Pr), 4.38 (ABXY, 1H, m, H-$3´_Y$), 5.97 (ABXY, 1H, m, H-$1´_X$), 6.88 (4H, AA´BB´, ^3J = 10.0 Hz, $H_{A,A´}$, aryl of DMT), 7.15 – 7.42 (11H, m, $H_{B,B´}$, aryl of DMT; H-3 of TPS; phenyl of DMT), 8.19 (1H, m, H-6). ^{13}C NMR (100 MHz, DMSO-d_6), thymine moiety: δ = 12.26 (5-CH_3), 109.35 (C-5), 140.23 (C-6), 150.46 (C-2), 163.74 (C-4); 2´-deoxyribose moiety: 39.41 (C-2´, hidden under the solvent), 61.33 (C-5´), 70.43 (C-3´), 79.90 (C-1´), 83.71 (C-4´); DMT: 55.00 (OCH_3), 87.24 (quat. C, DMT, aliphatic carbon), 112.76 (*o*-CH, aryl), CH (phenyl): 126.44, 127.41, 127.64; 128.91 (*m*-CH, aryl), 136.11 (*ipso*-C, phenyl); *ipso*-C, aryl: 146.79, 157.81; TPS: 23.84 (CH_3, *p*-*i*-Pr), 24.81 (CH_3, *o*-*i*-Pr), 28.02 (CH, *o*-*i*-Pr), 33.27 (CH, *p*-*i*-Pr), 121.37 (CH); *ipso*-C: 141.78, 147.25, 148.35; TPDMS: –3.20 (Si-CH_3), 17.80 (quat. C, Si-*t*-Bu), 25.80 (CH_3, Si-*t*-Bu). FT-IR (neat): $\tilde{\nu}$ = 3010, 2953, 2929, 2868, 1683, 1503, 1462, 988, 874 (cm^{-1}). FD-MS: *m/z* $[M]^+$ calcd for $C_{52}H_{68}N_2O_9SSi$: 924.4; found: 924.5.

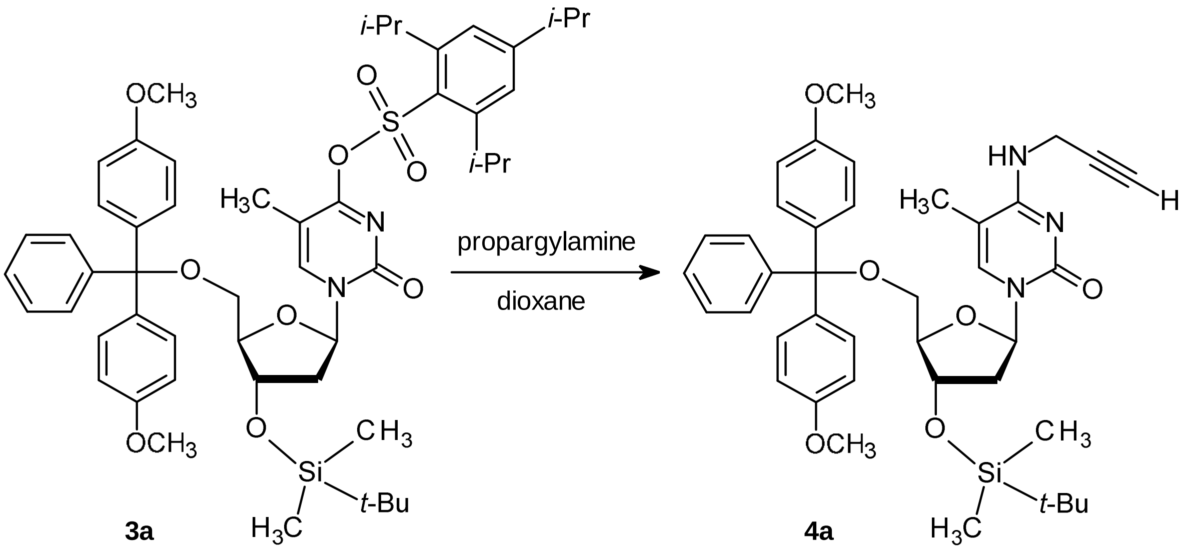

3a **4a**

3´-*O*-TBDMS-5´-*O*-DMT-5-methyl-*N*⁴-propargyl-2´-deoxycytidine (**4a**). To a stirred solution of **3a** (0.90 g, 0.97 mmol) in dry dioxane (15 mL), propargylamine (23.37 mmol, 1.5 mL, 24 equiv) was added dropwise and the reaction mixture was stirred at ambient temperature overnight. Distillation of the solvent and excess propargylamine under vacuum, and purification of the residue by CC (silica gel) with cyclohexane/EtOAc (2:1, containing 3% triethylamine) afforded a tan solid (0.07 g, 0.10 mmol, 10%, m.p. 75–78 ℃). TLC: silica gel, DCM/MeOH (20:1), R_f = 0.53. ^{1}H NMR (400 MHz, DMSO-d_6) δ = –0.06 (3H, s, Si-CH_3), –0.01 (3H, s, Si-CH_3), 0.78 (9H, s, Si-*t*-Bu), 1.54 (3H, m, 5-CH_3), 2.17 (ABXY, 2H, m, H-$2´_{A,B}$), 3.10 (ABXY, 1H, m, H-10_Y), 3.22 (ABX, 2H, m, $^2J_{AB}$ = 10.7 Hz, H-$5´_{A,B}$), 3.73 (6H, s, 2 x OCH_3), 3.82 (ABX, 1H, m, H-$4´_X$), 4.07 (ABXY, 2H, m, H-$8_{A,B}$), 4.43 (ABXY, 1H, m, H-$3´_Y$), 6.16 (ABXY, 1H, m, H-$1´_X$); DMT: aryl (AA´BB´, ^3J = 8.6 Hz, ^4J = 3.5 Hz): 6.89 (4H, $H_{A,A´}$), 7.26 (4H, $H_{B,B´}$), phenyl (AA´BB´C, ^3J = 7.5 Hz, H_C overlapped by $H_{B,B´}$ aryl): 7.31 (2H, $H_{B,B´}$), 7.39 (2H, $H_{A,A´}$); 7.58 (1H, m, H-6), 7.59 (ABXY, 1H, m, NH_X, partially overlapped by H-6). ^{13}C NMR (100 MHz, DMSO-d_6), thymine moiety: δ = 12.67 (5-CH_3), 101.75 (C-5), 135.34 (C-6), 154.61 (C-2), 162.35 (C-4); 2´-deoxyribose moiety: 40.70 (C-2´), 62.77 (C-5´), 71.45 (C-3´), 84.53 (C-1´), 85.93 (C-4´); propargyl group: 29.43 (C-8), 72.65 (C-10), 81.39 (C-9); DMT: 55.07 (OCH_3), 85.17 (quat. C, DMT, aliphatic carbon), 113.25 (*o*-CH, aryl), CH (phenyl): 126.85, 127.69, 127.91; 129.71 (*m*-CH, aryl), *ipso*-C (phenyl): 135.25, 137.39; *ipso*-C (aryl): 144.54, 158.21; TPDMS: –5.11, –4.78 (Si-CH_3); 17.63 (quat. C, Si-*t*-Bu), 25.62 (CH_3, Si-*t*-Bu). FT-IR (neat): $\tilde{\nu}$ = 3288, 3080, 2949, 1699, 1658, 1625, 1458, 1245, 1102, 824, 669 (cm^{-1}). FD-MS: *m/z* $[M]^+$ calcd for $C_{40}H_{49}N_3O_6Si$: 695.3; found: 695.6.

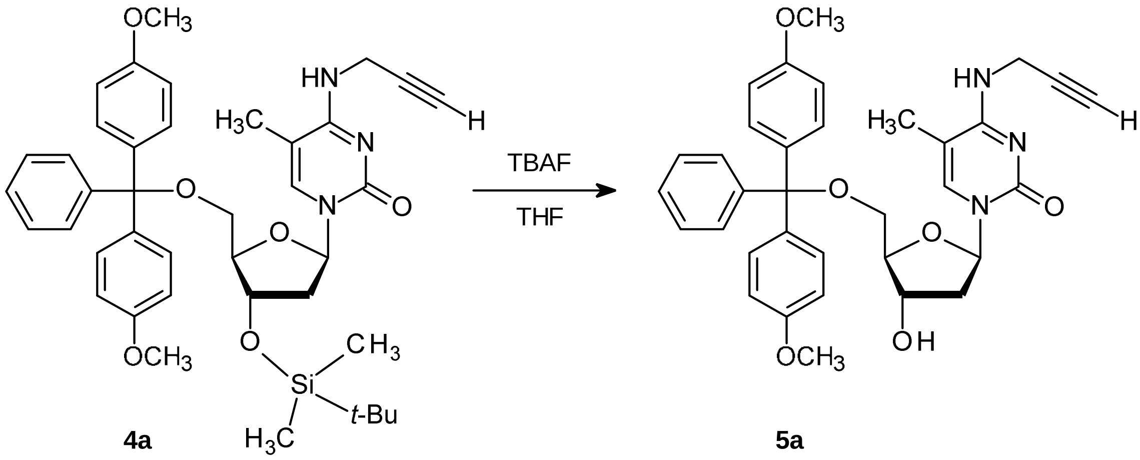

5´-*O*-DMT-5-methyl-*N*4-propargyl-2´-deoxycytidine (**5a**). Under Ar, TBAF (0.9 mL, 1M in THF, 0.9 mmol, 1.6 equiv) was added to a stirred solution of **4a** (0.40 g, 0.58 mmol) in anhydrous THF (2 mL). The solution was stirred at ambient temperature until TLC analysis indicated complete deprotection of the starting material (1 hour). EtOAc (50 mL) was added and the product mixture was extracted with saturated aqueous $NaHCO_3$ (3 x 30 mL). The organic layer was dried with anhydrous Na_2SO_4 and concentrated under vacuum. Purification of the residue by CC (aluminium oxide, basic) using the gradient system cyclohexane/EtOAc (1:2 → 1:3) → EtOAc/MeOH (5:1 → 4:1) yielded a brown solid (141.5 mg, 2.44 mmol, 42%, m.p. 95–100 °C). TLC: DCM/MeOH (15:1), R_f = 0.15. ^{1}H NMR (400 MHz, DMSO-d_6) δ = 1.45 (3H, m, 5-CH_3), 2.15 (ABXY, 2H, m, $^2J_{AB}$ = 13.3 Hz, H-2´$_{A,B}$), 3.00 (ABXY, 1H, m, H-10$_Y$), 3.18 (ABX, 2H, m, H-5´$_{A,B}$), 3.70 (6H, s, 2 x OCH_3), 3.87 (ABX, 1H, m, H-4´$_X$), 4.08 (ABXY, 2H, m, H-8$_{A,B}$), 4.30 (ABXY, 1H, m, H-3´$_Y$), 5.49 (1H, d, ^3J = 4.4 Hz, 3´-OH), 6.18 (ABXY, 1H, m, H-1´$_X$); DMT: aryl (AA´BB´, ^3J = 8.8 Hz): 6.87 (4H, $H_{A,A´}$), 7.23 (4H, $H_{B,B´}$); phenyl (AA´BB´C, phenyl, ^3J = 7.2 Hz, H_C overlapped by $H_{B,B´}$ aryl): 7.29 (2H, $H_{B,B´}$), 7.36 (2H, $H_{A,A´}$); 7.52 (1H, m, H-6), 7.61 (ABXY, 1H, m, NH_X). ^{13}C NMR (100 MHz, DMSO-d_6), thymine moiety: δ = 12.86 (5-CH_3), 102.57 (C-5), 135.87 (C-6), 155.45 (C-2), 162.81 (C-4); 2´-deoxyribose moiety: 40.94 (C-2´), 63.92 (C-5 ´), 70.93 (C-3´), 85.13 (C-1´), 86.30 (C-4´); propargyl group: 29.83 (C-8), 72.93 (C-10), 81.74 (C-9); DMT: 55.50 (OCH_3), 85.76 (quat. C, DMT, aliphatic carbon), 113.68 (*o*-CH, aryl), CH (phenyl): 127.35, 128.09, 128.41; 130.16 (*m*-CH, aryl), *ipso*-C (phenyl): 135.72, 137.81; *ipso*-C (aryl): 144.99, 158.57. FT-IR (neat): $\tilde{\nu}$ = 3305, 3280, 3080, 2953, 2249, 1658, 1605, 1417, 1376, 1249, 1339, 824, 681 (cm^{-1}). HR-MS (ESI): *m/z* $[M+Na]^+$ calcd for $C_{34}H_{35}N_3O_6Na$: 604.2423; found: 604.2419.

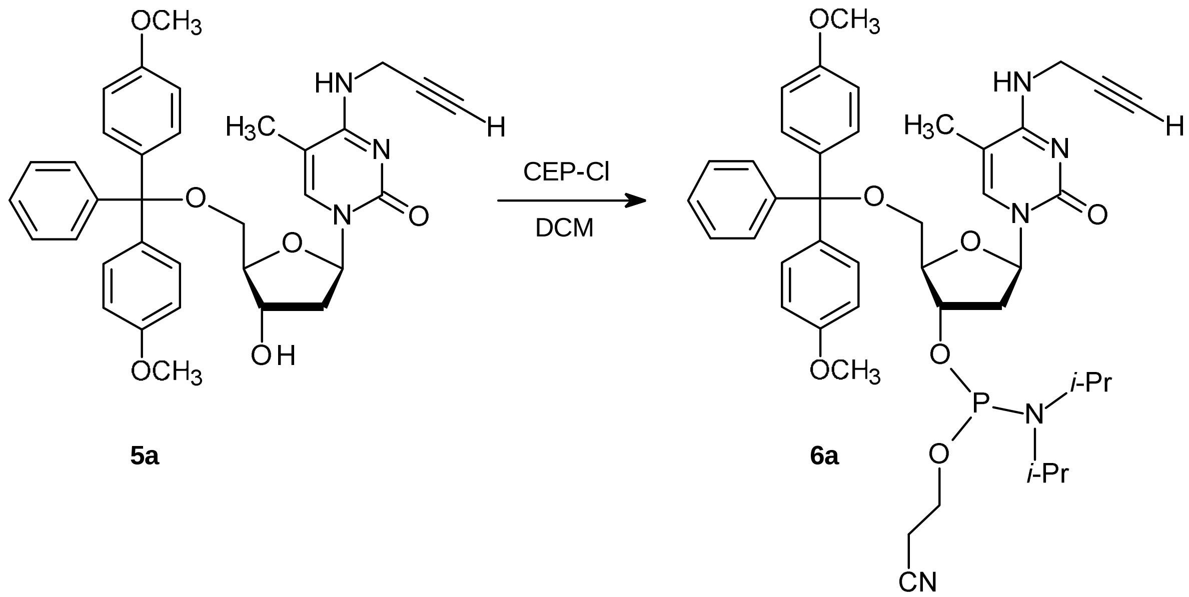

5a **6a**

3´-*O*-CEP-5´-*O*-DMT-5-methyl-*N*^{4}-propargyl-2´-deoxycytidine (**6a**). Under Ar, DIPEA (0.35 mL, 2.0 mmol, 5.0 equiv) was added to a stirred solution of **5a** (235 mg, 0.40 mmol) in anhydrous DCM (10 mL), followed by the dropwise addition of CEP-Cl (0.12 mL, 0.52 mmol, 1.3 equiv). The reaction mixture was stirred for 2 hours at ambient temperature. Aqueous $NaHCO_3$ (5%, 40 mL) was added and the product was extracted with DCM (3 x 20 mL). The organic layer was dried with anhydrous $MgSO_4$ and concentrated under vacuum. Purification of the pale yellow foam by CC (aluminium oxide, basic) with DCM/MeOH (70:1) yielded a colourless solid (162.5 mg, 2.08 mmol, 52%, m.p. 81–85 ℃), consisting of a mixture of the expected two diastereomers (dr = 50:50, ^{1}H and ^{31}P NMR). The product contained a larger amount of unknown impurities, presumably deriving from CEP-Cl (^{31}P NMR, δ = 14.40, 25.44, 31.33, 38.00). Furthermore, small amounts of trialkylphoshites (^{31}P NMR, δ = 138.78, 139.20) as well as the undesired *H* phosphonates (^{31}P NMR, δ = 7.73, 8.92) were formed during the synthesis and/or work-up procedures, i. e. prior to recording of the NMR spectra (compare with the ^{31}P NMR spectrum of compound **6b**). TLC: DCM/MeOH (15:1), R_f = 0.64, 0.69. ^{1}H NMR (400 MHz, DMSO-d_6) of both diastereomers: δ = 0.97, 1.093, 1.103, 1.124 (24H, d, ^3J = 6.75 Hz, CH_3, *i*-Pr); 1.51, 1.54 (6H, d, ^4J <0.8 Hz, 5-CH_3); 2.30 (ABXY, 4H, m, H-2´$_{A,B}$); 2.64, 2.76 (8H, t, ^3J = 5.6 Hz, CH_2CN); 3.10 (ABXY, 2H, m, H-10$_Y$), 3.26 (ABX, 4H, m, H-5´$_{A,B}$, partially overlapped by H_2O), 3.40 – 3.80 (6H, m, CH_2OP and CH of *i*-Pr, overlapped by impurity and OCH_3), 3.73 (12H, s, 2 x OCH_3); 3.99, 4.06 (ABX, 2H, m, H-4´$_X$, the latter resonance partially overlapped by H-8$_{A,B}$), 4.09 (ABXY, 4H, m, H-8$_{A,B}$), 4.51 (ABXY, 2H, m, H-3´$_Y$), 6.22 (ABXY, 2H, m, H-1´$_X$), DMT: aryl (AA´BB´, ^3J <9 Hz, ^4J = 2.1, 1.8 Hz): 6.876, 6.880 (8H, $H_{A,A´}$), aryl (AA´BB´, ^3J = 8.6, 8.8 Hz, ^4J = 2.4, 2.2 Hz): 7.242, 7.258 (8H, $H_{B,B´}$), phenyl (AA´BB´C, H_C overlapped by $H_{B,B´}$ aryl): 7.27 – 7.34 (4H, m, $H_{B,B´}$), 7.35 – 7.41 (4H, m, $H_{A,A´}$); 7.52, 7.55 (2H, q, ^4J <0.8 Hz, H-6); 7.606, 7.612 (ABXY, 2H, m, NH_X). ^{13}C NMR (100 MHz, DMSO-d_6) of both diastereomers: thymidine moiety: δ = 12.58 (5-CH_3, not resolved), 101.916 (C-5), 101.994 (C-5); 135.313 (C-6), 135.321 (C-6); 154.56 (C-2, not resolved), 162.32 (C-4, not resolved); 2´-deoxyribose moiety: 39.755 (C-2´, hidden under the solvent), 39.960

(C-2´, hidden under the solvent); 63.029 (C-5´), 63.202 (C-5´); 96.752 (d, $^{2}J_{PC}$ <2 Hz, C-3´), 96.828 (d, $^{2}J_{PC}$ <2 Hz, C-3´), 84.37 (d, $^{4}J_{PC}$ <3 Hz, C-1´, one resonance overlapped by C-4´), 84.58 (d, $^{3}J_{PC}$ <7 Hz, C-4´, not resolved); propargyl group: 29.43 (C-8), 72.71 (C-10), 81.33 (C-9); DMT: 55.06 (OCH_3, not resolved); quat. C (DMT, aliphatic carbon): 85.90, 85.97; 113.24 (*o*-CH, aryl, not resolved), CH (phenyl): 126.822, 126.846, 127.645, 127.691, 127.91 (not resolved); *m*-CH (aryl): 129.696, 129.732; *ipso*-C (phenyl): 135.149, 135.179, 137.31, 137.49; *ipso*-C (aryl, not resolved): 144.56, 158.18; phosphoramidite moiety: CH_2–C≡N: 19.74, 19.81 (d, $^{3}J_{PC}$ <6 Hz); *i*-Pr (d, $^{3}J_{PC}$ <7 Hz, CH_3): 24.185, 24.255, 24.285, 24.355; *i*-Pr (d, $^{2}J_{PC}$ <13 Hz, CH): 40.17, 42.54; CH_2–OP (d, $^{2}J_{PC}$ <3 Hz): 58.19, 58.38; quat. C (C≡N): 118.78, 118.98. ^{31}P NMR (162 MHz, DMSO-d_6) δ = 147.61, 147.97. FT-IR (neat): $\tilde{\nu}$ = 3288, 2929, 2157, 1662, 1629, 1605, 1462, 1249, 1332, 824, 694 (cm^{-1}). HR-MS (ESI): *m/z* $[M+Na]^+$ calcd for $C_{43}H_{52}N_5O_7PNa$: 804.3502; found: 804.3495.

Synthesis of 5-Methyl-*N*[4]-propargyl-2´-deoxycytidine (7a)

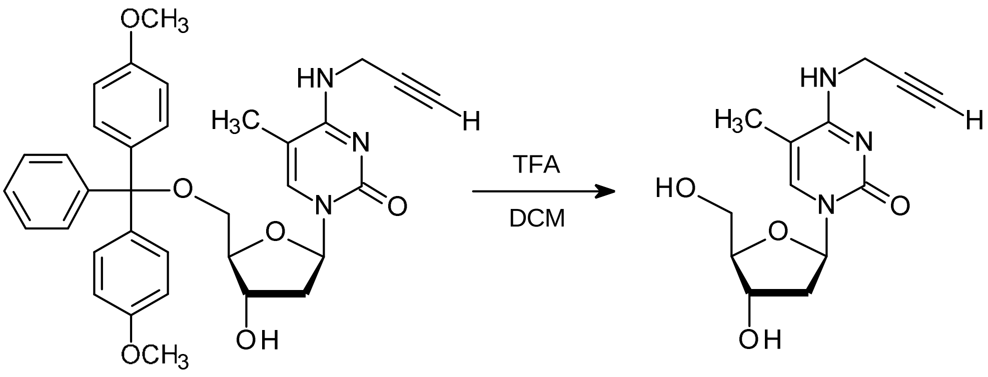

5a **7a**

5-Methyl-*N*^{4}-propargyl-2´-deoxycytidine (**7a**). A mixture of **5a** (0.24 g, 0.41 mmol) and TFA (63.0 µl, 0.83 mmol, 2.0 equiv) in DCM (5 mL) was stirred at ambient temperature until TLC showed complete deprotection of the starting material. Distillation of the solvent and excess TFA, and purification of the residue by CC (silica gel) with DCM/MeOH (15:1) gave a colourless solid (0.08 g, 0.29 mmol, 70%, m.p. 180 °C with decomposition). Furthermore, a sma ll amount of thymidine was isolated, probably due to the loss of propargylamine in the presence of acid and water. TLC: DCM/MeOH (6:1), R_f = 0.32. ^{1}H NMR (300 MHz, DMSO-d_6) δ = 1.84 (3H, m, 5-CH_3), 2.01 (ABXY, 2H, m, $^{2}J_{AB}$ = 13.2 Hz, H- 2´$_{A,B}$), 3.09 (ABXY, 1H, m, H-10$_Y$), 3.56 (ABX, 2H, m, H-5´$_{A,B}$), 3.75 (ABX, 1H, m, H-4´$_X$), 4.08 (ABXY, 2H, m, H-8$_{A,B}$), 4.20 (ABXY, 1H, m, H-3´$_Y$), 5.04 (1H, t, ^{3}J = 5.2 Hz, 5´-OH), 5.22 (1H, d, ^{3}J = 4.2 Hz, 3´-OH), 6.16 (ABXY, 1H, m, H-1´$_X$), 7.60 (ABXY, 1H, m, NH$_X$), 7.66 (1H, m, H-6). ^{13}C NMR (75 MHz, DMSO-d_6), thymine moiety: δ = 13.08 (5-CH_3), 101.55 (C-5), 149.38 (C-6), 154.81 (C-2), 162.33 (C-4); 2´-deoxyribose moiety: 40.35 (C-2´), 61.23 (C-5´), 70.19 (C-3´), 84.63 (C-1´), 87.50 (C-4´); propargyl group: 29.43 (C-8), 72.67 (C-10), 81.53 (C-9). FT-IR (neat): $\tilde{\nu}$ = 3280, 3114, 2929, 2866, 2275, 1711, 1679, 1609, 1335, 1200, 1180, 1049, 1021, 1000, 972, 824 (cm^{-1}). HR-MS (ESI): *m/z* $[M+Na]^+$ calcd for $C_{13}H_{17}N_3O_4Na$: 302.1116; found: 302.1127.

Synthesis of 3´-*O*-CEP-5´-*O*-DMT-N^4-propargyl-2´-deoxycytidine (**6b**)

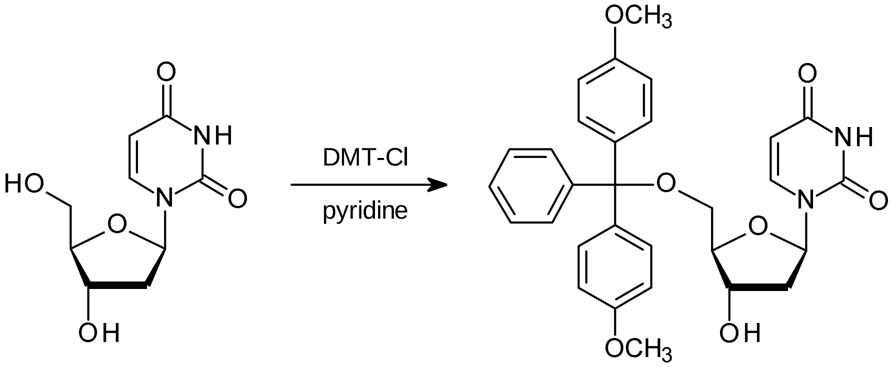

1b

5´-*O*-DMT-2´-deoxyuridine (**1b**). Under Ar, triethylamine (0.24 mL, 1.72 mmol, 0.78 equiv) was added to a stirred solution of 2´-deoxyuridine (0.50 g, 2.20 mmol) in dry pyridine (2.50 mL), followed by dropwise addition of DMT-Cl (1.12 g, 3.30 mmol, 1.50 equiv) in a minimal amount of dry pyridine. The reaction mixture was stirred at ambient temperature until all starting material has been converted to the tritylated product. MeOH (2 mL) was added and the resulting mixture was concentrated under vacuum. Purification of the residue by CC (silica gel) with DCM/MeOH (20:1, containing 1% triethylamine) yielded a colourless solid (0.96 g, 1.82 mmol, 83%, m.p. 98 °C). TLC: DCM/MeOH (15:1), R_f = 0.35. ^{1}H NMR (300 MHz, DMSO-d_6) δ = 2.18 (ABXY, 2H, m, H-2´$_{A,B}$), 3.20 (ABX, 2H, m, $^2J_{AB}$ = 10.4 Hz, H-5´$_{A,B}$), 3.74 (6H, s, 2 x OCH_3), 3.87 (ABX, 1H, m, H-4´$_X$), 4.28 (ABXY, 1H, m, H-3´$_Y$), 5.35 (1H, d, ^3J = 4.5 Hz, 3´-OH, partially overlapped by H-5), 5.37 (1H, d, ^3J = 8.1 Hz, H-5), 6.14 (ABXY, 1H, m, H-1´$_X$); DMT: aryl (AA´BB´, ^3J = 8.6 Hz): 6.89 (4H, $H_{A,A´}$), 7.24 (4H, $H_{B,B´}$), phenyl (AA´BB´C, ^3J = 7.6 Hz, H_C overlapped by $H_{B,B´}$ aryl): 7.31 (2H, $H_{B,B´}$), 7.37 (2H, $H_{A,A´}$); 7.64 (1H, d, ^2J = 8.1 Hz, H-6), 11.33 (1H, s, NH). ^{13}C NMR (100 MHz, DMSO-d_6), uracil moiety: δ = 101.55 (C-5), 140.44 (C-6), 150.32 (C-2), 163.03 (C-4), 2´-deoxyribose moiety: 39.70 (C-2´, hidden under the solvent), 63.44 (C-5´), 69.95 (C-3´), 84.14 (C-1´), 85.36 (C-4´); DMT: 55.03 (OCH_3); 85.77 (quat. C, DMT, aliphatic carbon), 113.22 (*o*-CH, aryl), CH (phenyl): 126.75, 127.69, 127.88; 129.75 (*m*-CH, aryl), *ipso*-C (phenyl): 135.22, 135.43; *ipso*-C (aryl): 144.71, 158.10. FT-IR (neat): $\tilde{\nu}$ = 3056, 2970, 2929, 2831, 1679, 1605, 1511, 1458, 1376, 1245, 1176, 1090, 1029, 824 (cm^{-1}). FD-MS: *m/z* $[M+Na]^+$ calcd for $C_{30}H_{30}N_2O_7Na$: 530.2; found: 530.3.

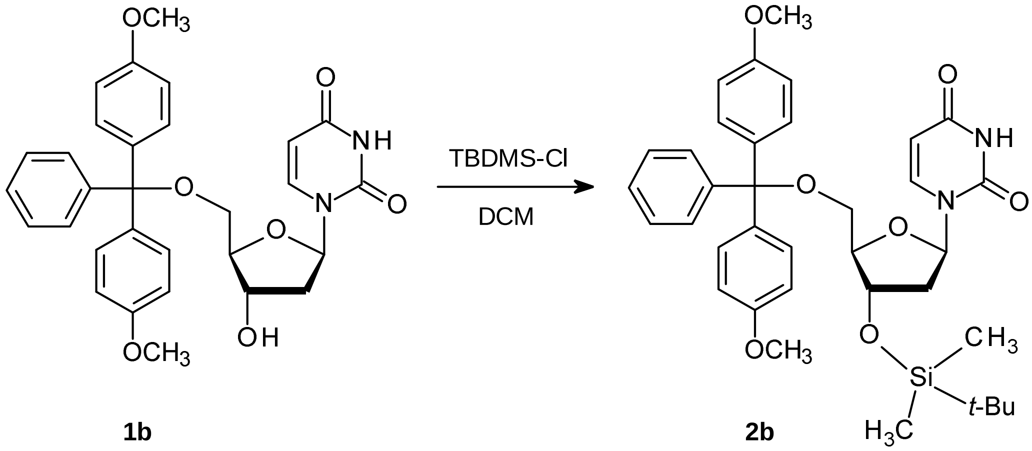

1b **2b**

3´-*O*-TBDMS-5´-*O*-DMT-2´-deoxyuridine (**2b**). Under Ar, imidazole (0.34 g, 5.0 mmol, 4.4 equiv) was added to a stirred solution of **1b** (0.60 g, 1.13 mmol) in dry DMF (6 mL), followed by the dropwise addition of TBDMS-Cl (0.38 g, 2.49 mmol, 2.2 equiv) in a minimal amount of dry DCM. The reaction mixture was stirred at ambient temperature until TLC indicated complete consumption of the starting material (12 hours). MeOH (1 mL) was added and the solution was poured into EtOAc (30 mL). The organic layer was extracted with 5% aqueous $NaHCO_3$ (2 x 30 mL), and washed with water (2 x 20 mL) and brine (1 x 30 mL). Drying of the organic layer with anhydrous $MgSO_4$ and evaporation of the solvent afforded a colourless solid (0.71 g, 1.10 mmol, 98%, m.p. 87–89 °C). TLC: silica gel, cyclohexane/EtOAc (3:1), R_f = 0.14. ^{1}H NMR (300 MHz, DMSO-d_6) δ = –0.05 (3H, s, Si-CH_3), 0.00 (3H, s, Si-CH_3), 0.78 (9H, s, Si-*t*-Bu), 2.23 (ABXY, 2H, m, H-2´$_{A,B}$), 3.25 (ABX, 2H, m, $^2J_{AB}$ = 10.3 Hz, H-5´$_{A,B}$), 3.73 (6H, s, 2 x OCH_3), 3.81 (ABX, 1H, m, H-4´$_X$), 4.43 (ABXY, 1H, m, H-3´$_Y$), 5.43 (1H, d, ^3J = 8.1 Hz, H-5), 6.15 (ABXY, 1H, m, H-1´$_X$); DMT: aryl (AA´BB´, ^3J = 8.4 Hz): 6.88 (4H, $H_{A,A´}$), 7.26 (4H, $H_{B,B´}$), phenyl (AA´BB´C, ^3J = 7.6 Hz, H_C overlapped by $H_{B,B´}$ aryl): 7.31 (2H, $H_{B,B´}$), 7.37 (2H, $H_{A,A´}$); 7.75 (1H, d, ^3J = 8.1 Hz, H-6), 11.37 (1H, s, NH). ^{13}C NMR (100 MHz, DMSO-d_6), uracil moiety: δ = 101.64 (C-5), 140.58 (C-6), 150.30 (C-2), 163.07 (C-4); 2´-deoxyribose moiety: 39.55 (C-2´, hidden under the solvent), 62.63 (C-5´), 70.95 (C-3´), 84.07 (C-1´), 84.99 (C-4´), DMT: 55.00 (OCH_3), 85.93 (quat. C, DMT, aliphatic carbon), 113.17 (*o*-CH, aryl); phenyl (CH): 126.77, 127.71, 127.83; 129.73 (*m*-CH, aryl), *ipso*-C (phenyl): 135.17, 135.29; *ipso*-C (aryl): 144.60, 158.17; TPDMS: –5.21, –4.84 (Si-CH_3), 17.55 (quat. C, Si-*t*-Bu), 25.55 (CH_3, Si-*t*-Bu). FT-IR (neat): $\tilde{\nu}$ = 3060, 2945, 2925, 2855, 1687, 1605, 1507, 1462, 1388, 1249, 1172, 1102, 1033, 824 (cm^{-1}). FD-MS: *m/z* $[M]^+$ calcd for $C_{36}H_{44}N_2O_7Si$: 644.3; found: 644.3.

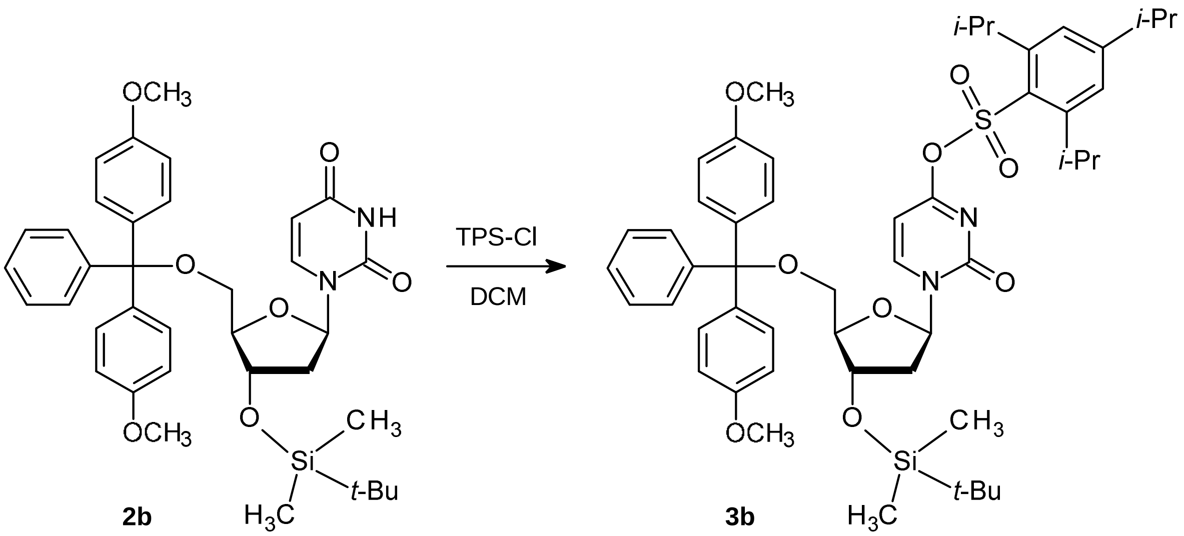

3´-*O*-TBDMS-5´-*O*-DMT-*O*^{4}-TPS-2´-deoxyuridine (**3b**). To a stirred solution of **2b** (2.00 g, 3.10 mmol) in dry DCM (2 mL), solutions of DMAP (0.03 g, 0.28 mmol, 0.09 equiv) and triethylamine (2.16 mL, 15.50 mmol, 5.00 equiv) in a minimal amount of DCM were added, whereas the total reaction volume did not exceed 4 mL. Subsequently, a solution of TPS-Cl (1.13 g, 3.72 mmol, 1.20 equiv) in a minimal amount of dry DCM was added dropwise and the reaction mixture was stirred at ambient temperature overnight. The resulting mixture was concentrated under vacuum, triethylammonium chloride was precipitated with petroleum ether and the precipitate was removed by filtration. Concentration of the filtrate under vacuum and purification of the residue by CC using the gradient system cyclohexane/EtOAc (15:1 → 9:1, containing 1% triethylamine) yielded a colourless solid (1.49 g, 1.64 mmol, 53%, m.p. 84–87 ℃). Furthermore, a smal l amount (100 mg, 3.5%, ^{1}H NMR) of the regioisomer of **3b** was isolated, bearing the TPS group at the C-2 oxygen (the compound was neither isolated nor thoroughly characterised). TLC: silica gel, cyclohexane/EtOAc (3:1), R_f = 0.70. ^{1}H NMR (400 MHz, DMSO-*d*$_6$) δ = –0.06 (3H, s, Si-CH$_3$), –0.01 (3H, s, Si-CH$_3$), 0.77 (9H, s, Si-*t*-Bu), 1.11 (12H, d, ^{3}J = 6.8 Hz, CH$_3$, *o*-*i*-Pr), 1.16 (6H, d, ^{3}J = 6.8 Hz, CH$_3$, *p*-*i*-Pr), 2.22 (ABXY, 2H, m, H-2´$_{A,B}$), 2.80 (1H, sept, ^{3}J = 6.8 Hz, CH, *p*-*i*-Pr), 3.21 (ABX, 2H, m, ^{3}J = 10.7 Hz, H-5´$_{A,B}$), 3.73 (6H, s, 2 x OCH$_3$), 3.79 (ABX, 1H, m, H-4´$_X$, partially overlapped by OCH$_3$), 4.41 (ABXY, 1H, m, H-3´$_Y$), 4.55 (2H, sept, ^{3}J = 6.8 Hz, CH, *o*-*i*Pr), 5.42 (1H, d, ^{3}J = 8.0 Hz, H-5), 6.12 (ABXY, 1H, m, H-1´$_X$); DMT and TPS: aryl DMT (AA´BB´, ^{3}J = 8.5 Hz): 6.89 (4H, H$_{A,A´}$), 7.24 (4H, H$_{B,B´}$), 7.15 – 7.40 (7H, m, phenyl DMT and H-3 TPS); 7.72 (1H, d, ^{3}J = 8.0 Hz, H-6). ^{13}C NMR (100 MHz, DMSO-*d*$_6$), uracil moiety: δ = 99.50 (C-5), 140.21 (C-6), 150.42 (C-2), 163.04 (C-4); 2´-deoxyribose moiety: 39.83 (C-2´, hidden under the solvent), 60.85 (C-5´), 69.18 (C-3´), 79.88 (C-1´), 83.74 (C-4´), DMT: 54.98 (OCH$_3$), 85.89 (quat. C, DMT, aliphatic carbon), 112.74 (*o*-CH, aryl); CH (phenyl): 126.41, 127.39, 127.62; 128.89 (*m*-CH, aryl), 135.16 (*ipso*-C, phenyl); *ipso*-C (aryl): 146.77, 157.78; TPS: 23.83 (CH$_3$, *o*-*i*-Pr), 24.80 (CH$_3$, *p*-*i*-Pr), 28.02 (CH, *o*-*i*-Pr), 33.26 (CH, *p*-*i*-Pr), 121.35 (CH); *ipso*-C: 141.71, 147.24, 148.33; TPDMS: –5.17, –4.82 (Si-CH$_3$), 17.57 (quat. C, Si-*t*-Bu), 25.68 (CH$_3$, Si-*t*-Bu). FT-IR (neat): $\tilde{\nu}$ = 2953, 2925, 2851, 1683, 1621, 1605, 1635, 1503, 1458, 1384, 1274, 1172, 1102, 1029, 1000, 829 (cm^{-1}). MS (MALDI-TOF): *m/z* [M+K]$^+$ calcd for $C_{51}H_{66}N_2O_9SSiK$: 949.39; found: 949.47.

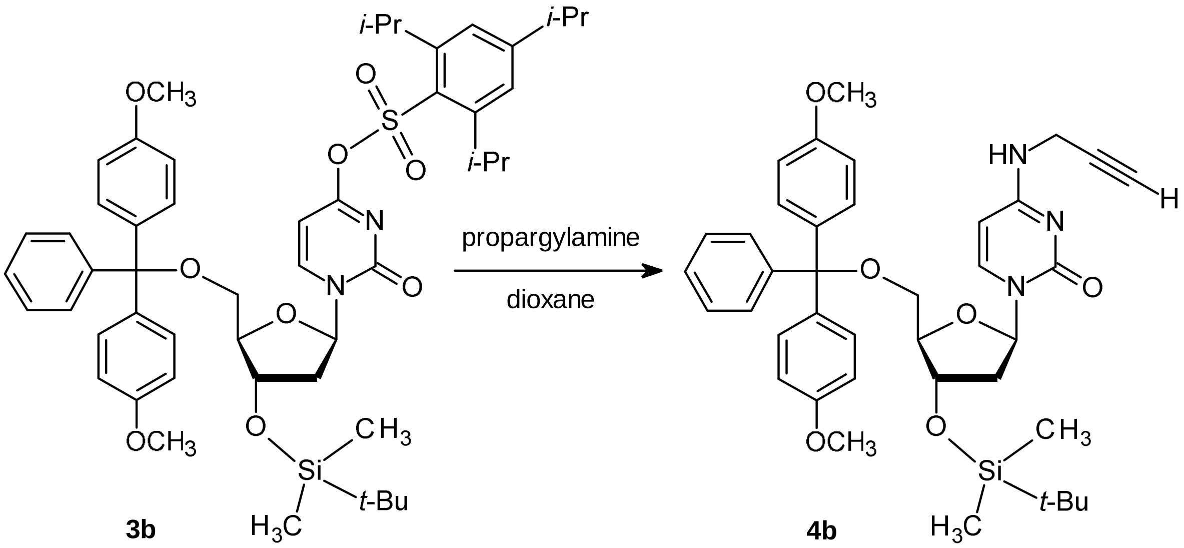

3´-*O*-TPDMS-5´-*O*-DMT-*N*[4]-propargyl-2´-deoxycytidine (**4b**). To a stirred solution of **3b** (0.30 g, 0.33 mmol) in dry dioxane (5 mL), propargylamine (0.21 mL, 3.30 mmol, 10.0 equiv) was added dropwise and the reaction mixture was stirred at ambient temperature overnight. Distillation of the solvent and excess propargylamine under vacuum, and purification of the residue by CC (aluminium oxide, basic) using the gradient system cyclohexane/EtOAc (1:2 → 1:3) → EtOAc/MeOH (5:1 → 1:2) afforded a brown solid (0.07 g, 0.11 mmol, 33%, m.p. 81–83 °C). TLC: silica gel, DCM/MeOH (15:1), R_f = 0.54. ^{1}H NMR (300 MHz, DMSO-d_6) δ = –0.07 (3H, s, Si-CH_3), –0.01 (3H, s, Si-CH_3), 0.77 (9H, s, Si-*t*-Bu), 2.15 (ABXY, 2H, m, H-$2´_{A,B}$), 3.23 (ABX, 2H, m, H-$5´_{A,B}$, partially overlapped by both H-10 and H_2O), 3.18 (ABXY, 1H, m, H-10_Y, partially overlapped by H-$5´_{A,B}$), 3.74 (6H, s, 2 x OCH_3), 3.80 (ABX, 1H, m, H-$4´_X$), 4.07 (ABXY, 2H, m, H-$8_{A,B}$), 4.39 (ABXY, 1H, m, H-$3´_Y$), 5.64 (1H, d, ^3J = 7.4 Hz, H-5), 6.13 (ABXY, 1H, m, H-$1´_X$); DMT: aryl (AA´BB´, ^3J = 8.4 Hz): 6.89 (4H, $H_{A,A´}$), 7.25 (4H, $H_{B,B´}$), phenyl (AA´BB´C, ^3J = 7.8 Hz, H_C overlapped by $H_{B,B´}$ aryl): 7.32 (2H, $H_{B,B´}$), 7.37 (2H, $H_{A,A´}$); 7.78 (1H, d, ^3J = 7.4 Hz, H-6), 8.09 (ABXY, 1H, m, NH_X). ^{13}C NMR (100 MHz, DMSO-d_6), uracil moiety: δ = 99.50 (C-5), 140.15 (C-6), 149.30 (C-2), 162.86 (C-4); 2´-deoxyribose moiety: 38.60 (C-2´), 62.39 (C-5´), 70.82 (C-3´), 84.61 (C-1´), 84.88 (C-4´); propargyl group: 29.03 (C-8), 73.43 (C-10), 80.80 (C-9); DMT: 55.05 (OCH_3), 85.88 (quat. C, DMT, aliphatic carbon), 113.20 (*o*-CH, aryl); CH (phenyl): 126.80, 127.69, 127.86; 129.72 (*m*-CH, aryl), *ipso*-C (phenyl): 135.20, 135.24; *ipso*-C (aryl): 144.49, 158.14; TPDMS: –5.18, –4.80 (Si-CH_3), 17.59 (quat. C, Si-*t*-Bu), 25.59 (CH_3, Si-*t*-Bu). FT-IR (neat): $\tilde{\nu}$ = 3284, 2950, 2925, 2851, 1635, 1604, 1578, 1507, 1459, 1443, 1295, 1248, 1174, 1103, 1029, 828 (cm^{-1}). MS (MALDI-TOF): *m/z* $[M+K]^+$ calcd for $C_{39}H_{47}N_3O_6SiK$: 720.29; found: 720.32.

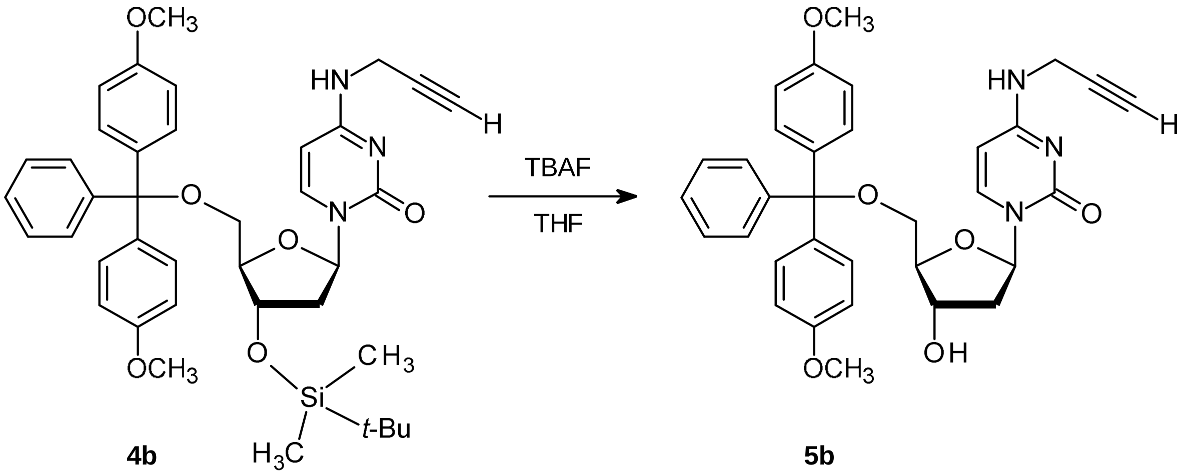

4b **5b**

5′-*O*-DMT-*N*[4]-propargyl-2′-deoxycytidine (**5b**). Under Ar, TBAF (0.34 mL, 1M in THF, 1.6 equiv, 0.34 mmol) was added to a stirred solution of **4b** (0.14 g, 0.21 mmol) in anhydrous THF (5 mL). The reaction mixture was stirred at ambient temperature until TLC analysis indicated complete deprotection of the starting material (1 hour). EtOAc (20 mL) was added and the mixture was washed with saturated aqueous $NaHCO_3$ (3 x 10 mL). The organic layer was dried with anhydrous Na_2SO_4 and concentrated under vacuum. Purification of the residue by CC (aluminium oxide, basic), using the gradient system cyclohexane/EtOAc (1:2 → 1:3) → EtOAc/MeOH (5:1 → 1:2) afforded a brown solid (0.11 g, 0.19 mmol, 91%, m.p. 123–125 °C). TLC: sil ica gel, DCM/MeOH (15:1), R_f = 0.24. ^{1}H NMR (300 MHz, DMSO-d_6) δ = 2.11 (ABXY, 2H, m, H-2′$_{A,B}$), 3.17 (ABXY, 1H, m, H-10$_Y$, partially overlapped by H-5′$_{A,B}$), 3.20 (ABX, 2H, m, H-5′$_{A,B}$, partially overlapped by H-10), 3.74 (6H, s, 2 x OCH_3), 3.87 (ABX, 1H, m, H-4′$_X$), 4.06 (ABXY, 2H, m, H-8$_{A,B}$), 4.25 (ABXY, 1H, m, H-3′$_Y$), 5.39 (1H, m, 3′-OH), 5.62 (1H, d, ^3J = 7.4 Hz, H-5), 6.16 (ABXY, 1H, m, H-1′$_X$); DMT: aryl (AA′BB′, ^3J = 8.4 Hz): 6.89 (4H, $H_{A,A'}$), 7.22 (4H, $H_{B,B'}$), phenyl (AA′BB′C, ^3J = 7.9 Hz, H_C overlapped by $H_{B,B'}$ aryl): 7.31 (2H, $H_{B,B'}$), 7.38 (2H, $H_{A,A'}$); 7.67 (1H, d, ^3J = 7.4 Hz, H-6), 8.15 (ABXY, 1H, m, NH_X). ^{13}C NMR (100 MHz, DMSO-d_6), uracil moiety: δ = 99.53 (C-5), 140.09 (C-6), 154.71 (C-2), 162.86 (C-4); 2′-deoxyribose moiety: 39.64 (C-2′, hidden under the solvent), 63.33 (C-5′), 69.90 (C-3′), 84.74 (C-1′), 85.21 (C-4′); propargyl group: 29.04 (C-8), 73.42 (C-9), 80.86 (C-10); DMT: 55.07 (OCH_3), 85.76 (quat. C, DMT, aliphatic carbon), 113.24 (*o*-C, aryl), CH (phenyl): 126.78, 127.71, 127.90; 129.74 (*m*-C, aryl), *ipso*-C (phenyl): 135.31, 135.43; *ipso*-C (aryl): 144.72, 158.11. FT-IR (neat): $\tilde{\nu}$ = 3276, 2925, 2835, 1638, 1572, 1458, 1442, 1298, 1245, 1168, 1082, 1029, 824 (cm^{-1}). MS (MALDI-TOF): *m/z* $[M]^+$ calcd for $C_{33}H_{33}N_3O_6$: 567.2; found: 567.3.

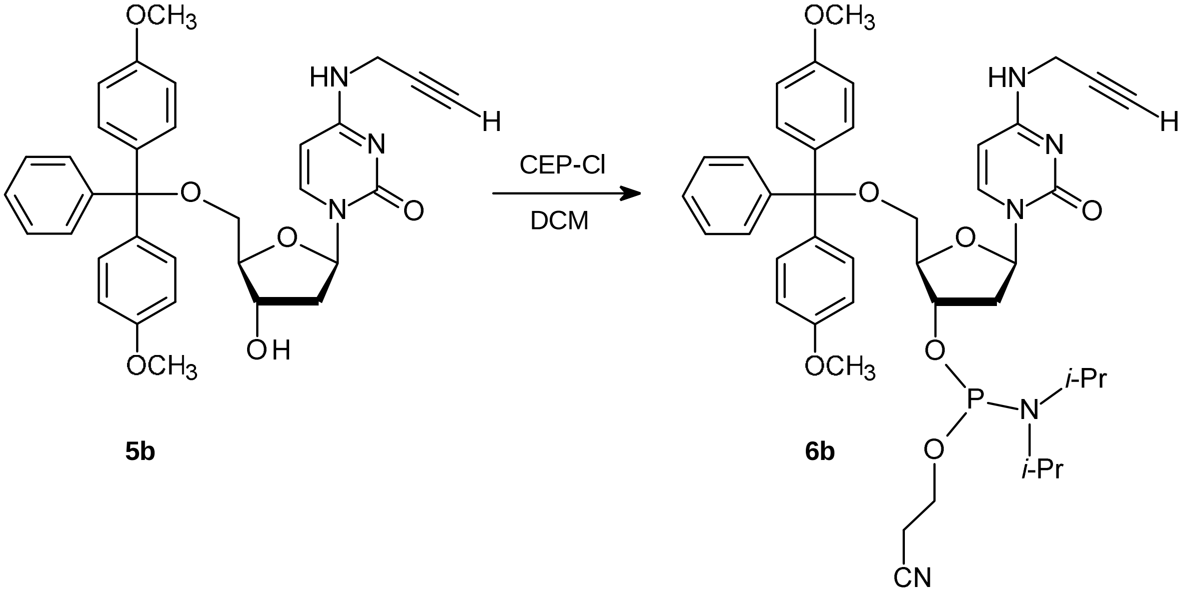

3´-*O*-CEP-5´-*O*-DMT-*N*^{4}-propargyl-2´-deoxycytidine (**6b**). Following a consecutive azeotropic dehydration of **5b** (0.15 g, 0.26 mmol) with pyridine, toluene and DCM, **5b** was dissolved in anhydrous DCM (5 mL) and placed under Ar. DIPEA (0.23 mL, 1.32 mmol, 5.0 equiv) was added to the stirred solution, followed by the dropwise addition of CEP-Cl (0.09 mL, 0.39 mmol, 1.5 equiv). The reaction mixture was stirred for 3 hours at ambient temperature. 5% Aqueous $NaHCO_3$ (10 mL) was added and the product was extracted with DCM (3 x 15 mL). The organic layer was dried with anhydrous $MgSO_4$ and concentrated under vacuum. Purification of the brown foam by CC (aluminium oxide, basic) with DCM/MeOH (70:1) yielded a tan oil (0.12 g, 0.16 mmol, 60%), consisting of a mixture of two diastereomers (dr = 85:15, ^{1}H NMR). TLC: DCM/MeOH (15:1), R_f = 0.45, 0.49. ^{1}H NMR (400 MHz, DMSO-d_6; only the resonances of the major diastereomer are indicated): δ = 0.98, 1.10 (12H, d, ^3J = 6.6 Hz, CH_3, *i*-Pr), 2.29 (ABXY, 2H, m, H-2´$_{A,B}$), 2.76 (2H, t, ^3J = 5.8 Hz, CH_2CN), 3.18 (ABXY, 1H, m, H-10$_Y$), 3.25 (ABX, 2H, m, H-5´$_{A,B}$), 3.43 – 3.79 (4H, m, CH_2PO and CH of *i*-Pr, partially hidden under OCH_3), 3.73 (6H, s, 2 x OCH_3), 3.99 (ABX, 1H, m, H-4´$_X$), 4.07 (ABXY, 2H, m, H-8$_{A,B}$), 4.49 (ABXY, 1H, m, H-3´$_Y$), 5.63 (2H, d, ^3J = 7.3 Hz, H-5), 6.18 (ABXY, 2H, m, H-1´$_X$); DMT: aryl (AA´BB´, ^3J = 8.4 Hz): 6.88 (4H, $H_{A,A´}$), 7.24 (4H, $H_{B,B´}$), phenyl (AA´BB´C, ^3J = 7.7 Hz, H_C overlapped by $H_{B,B´}$ aryl): 7.30 (2H, $H_{B,B´}$), 7.37 (2H, $H_{A,A´}$); 7.70 (1H, d, ^3J = 7.3 Hz, H-6), 8.10 (ABXY, 1H, m, NH$_X$). ^{13}C NMR (100 MHz, DMSO-d_6; only the resonances of the major diastereomer are indicated), uracil moiety: δ = 94.42 (C-5), 140.28 (C-6), 154.60 (C-2), 158.14 (C-4); 2´-deoxyribose moiety: 39.67 (C-2´, partially overlapped by solvent), 62.93 (C-5´), 95.28 (d, $^2J_{PC}$ <2 Hz, C-3´), 84.86 (C-1´), 85.84 (C-4´); propargyl group: 29.06 (C-8), 73.48 (C-10), 80.77 (C-9); DMT: 55.05 (OCH_3), 84.85 (quat. C, DMT, aliphatic carbon), 113.21 (*o*-C, aryl), CH (phenyl): 126.79, 127.66, 127.87; 129.71 (*m*-C, aryl), *ipso*-C (phenyl): 135.17, 135.28; *ipso*-C (aryl): 144.59, 162.85; phosphoramidite moiety: 19.80 (d, $^3J_{PC}$ = 7.0 Hz, CH_2–C≡N); *i*-Pr (d, $^3J_{PC}$ = 7.3 Hz, CH_3): 24.289, 24.191; 42.57 (d, $^2J_{PC}$ = 12.2 Hz, CH, *i*-Pr), 58.37 (d, $^2J_{PC}$ <1 Hz, CH_2–OP), 118.97 (quat. C, C≡N). ^{31}P NMR (162 MHz, DMSO-d_6) δ = 148.79 (major diastereomer), 148.38 (minor diastereomer). FT-IR (neat): $\tilde{\nu}$ = 3280, 3084, 3031,

2962, 2921, 2863, 2353, 2332, 2246, 1638, 1572, 1503, 1459, 1437, 1300, 1250, 1170, 1117, 1068, 1026, 973, 824 (cm^{-1}). HR-MS (ESI): *m/z* [M-H]$^{-}$ calcd for $C_{42}H_{51}N_5O_7P$: 768.3526; found: 768.3522.

Synthesis of N^4-Propargyl-2´-deoxycytidine (7b)

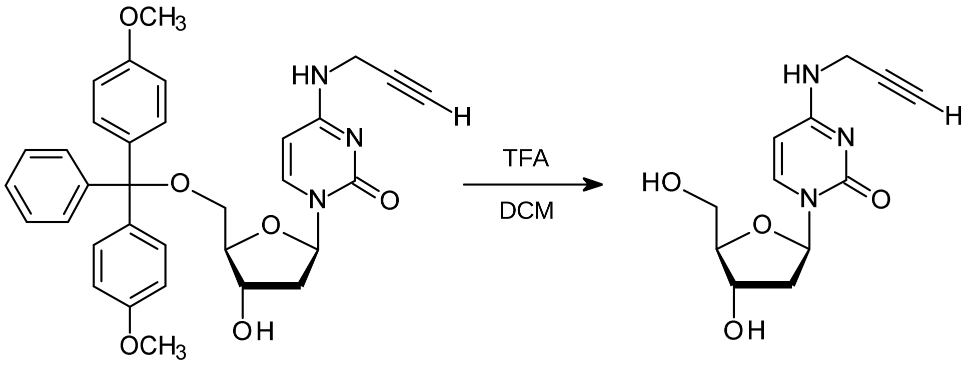

5b **7b**

N^4-Propargyl-2´-deoxycytidine (**7b**). A mixture of **5b** (0.10 g, 0.18 mmol) and TFA (20 µl, 0.26 mmol, 1.5 equiv) in DCM (5 mL) was stirred at ambient temperature until TLC analysis showed complete deprotection of the starting material. Distillation of the solvent and excess TFA, and purification of the residue by CC with DCM/MeOH (15:1) yielded a beige-coloured solid (0.03 g, 0.12 mmol, 70%, m.p. 170–175 °C). TLC: DCM/MeOH (5:1), R_f = 0.27. ^{1}H NMR (300 MHz, DMSO-d_6) δ = 2.02 (ABXY, 2H, m, $^2J_{AB}$ = 13.1 Hz, H-2´$_{A,B}$), 3.16 (ABXY, 1H, m, H-10$_Y$), 3.54 (ABX, 2H, m, H-5´$_{A,B}$), 3.76 (ABX, 1H, m, H-4´$_X$), 4.05 (ABXY, 2H, m, H-8$_{A,B}$), 4.19 (ABXY, 1H, m, H-3´$_Y$), 4.99 (1H, m, 5´-OH), 5.20 (1H, d, ^3J = 3.9 Hz, 3´-OH), 5.78 (1H, d, ^3J = 7.5 Hz, H-5), 6.14 (ABXY, 1H, m, H-1´$_X$), 7.81 (1H, d, ^3J = 7.5 Hz, H-6), 8.09 (ABXY, 1H, m, NH$_X$). ^{13}C NMR (75 MHz, DMSO-d_6), uracil moiety: δ = 94.67 (C-5), 140.48 (C-6), 154.87 (C-2), 162.90 (C-4); 2´-deoxyribose moiety: 40.39 (C-2´, partially overlapped by solvent), 61.32 (C-5´), 70.36 (C-3´), 84.95 (C-1´), 87.26 (C-4´); propargyl group: 29.05 (C-8), 73.39 (C-10), 80.89 (C-9). FT-IR (neat): $\tilde{\nu}$ = 3375, 2975, 2931, 2874, 2357, 2325, 1808, 1672, 1563, 1521, 1448, 1376, 1308, 1182, 1130, 1049, 945, 845 (cm^{-1}). HR-MS (ESI): *m/z* [M+Na]$^+$ calcd for $C_{12}H_{15}N_3O_4Na$: 288.0960; found: 288.0971.

Spectra of 3´-*O*-CEP-5´-*O*-DMT-5-methyl-N^4-propargyl-2´-deoxycytidine (6a)

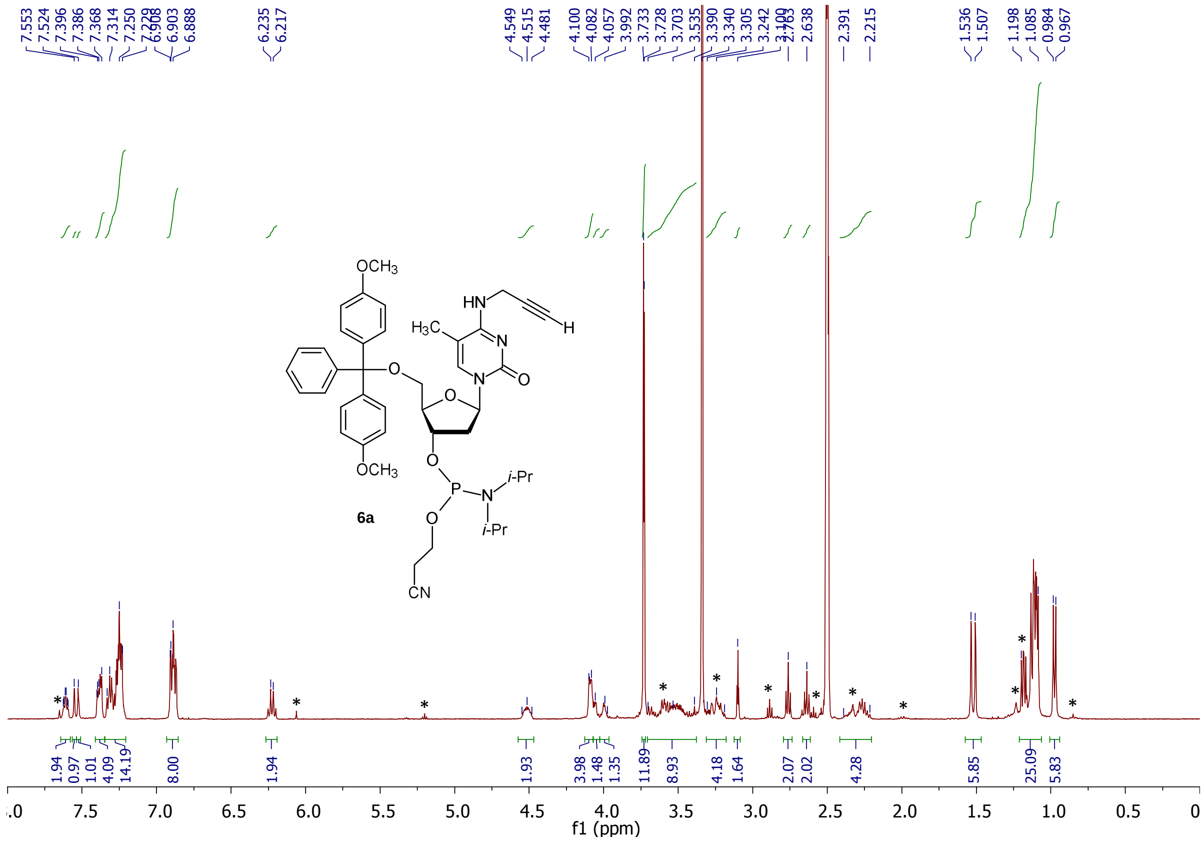

Figure S1. ^{1}H NMR spectrum (400 MHz, DMSO-d_6) of 3´-*O*-CEP-5´-*O*-DMT-5-methyl-N^4-propargyl-2´-deoxycytidine (**6a**).

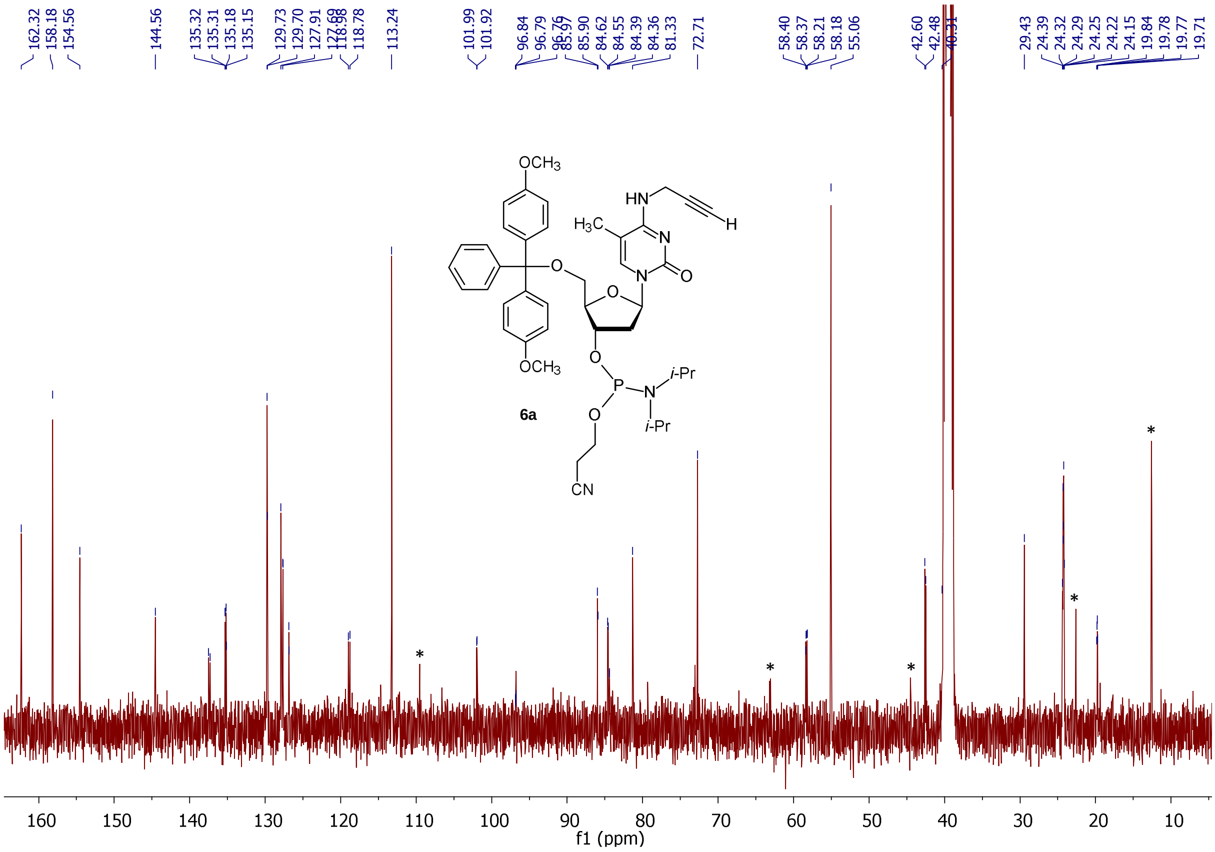

Figure S2. ^{13}C NMR spectrum (100 MHz, DMSO-d_6) of 3´-*O*-CEP-5´-*O*-DMT-5-methyl-N^4-propargyl-2´-deoxycytidine (**6a**).

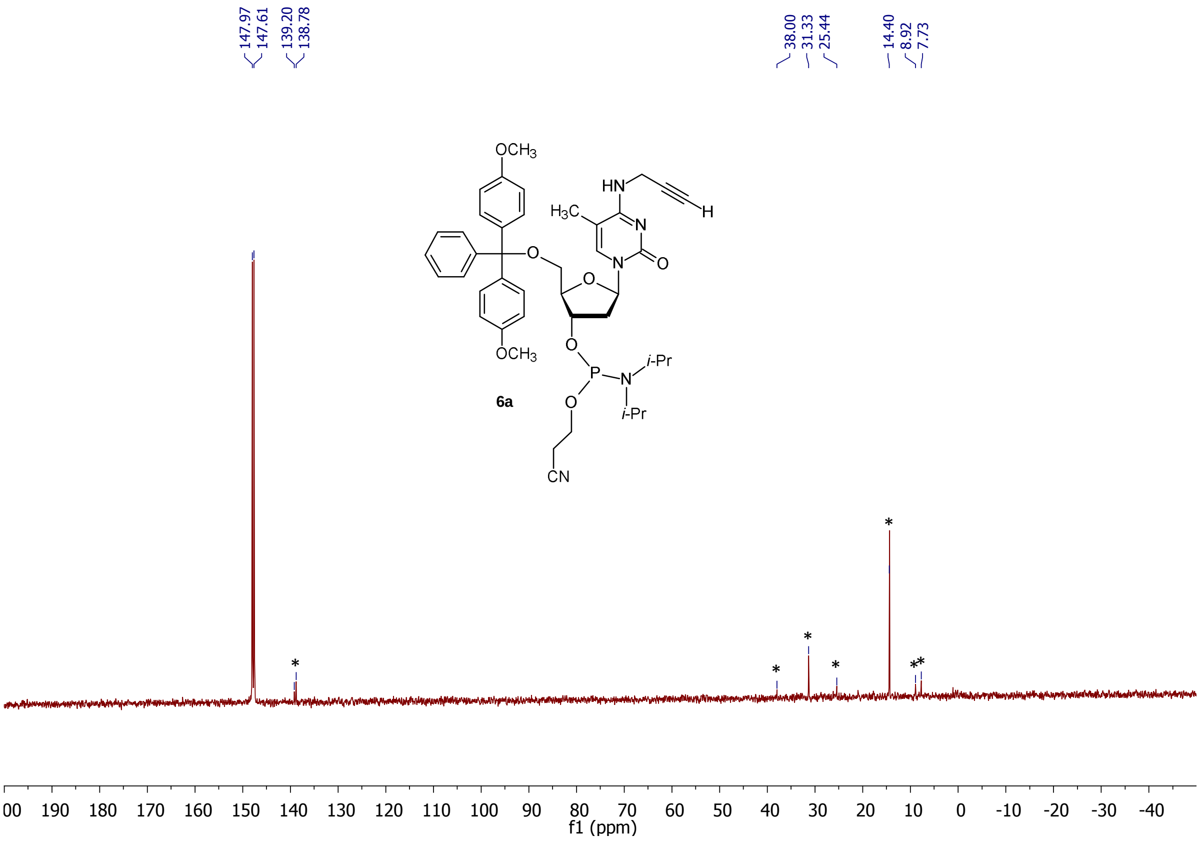

Figure S3. ^{31}P NMR spectrum (162 MHz, DMSO-d_6) of 3´-*O*-CEP-5´-*O*-DMT-5-methyl-*N*[4]-propargyl-2´-deoxycytidine (**6a**).

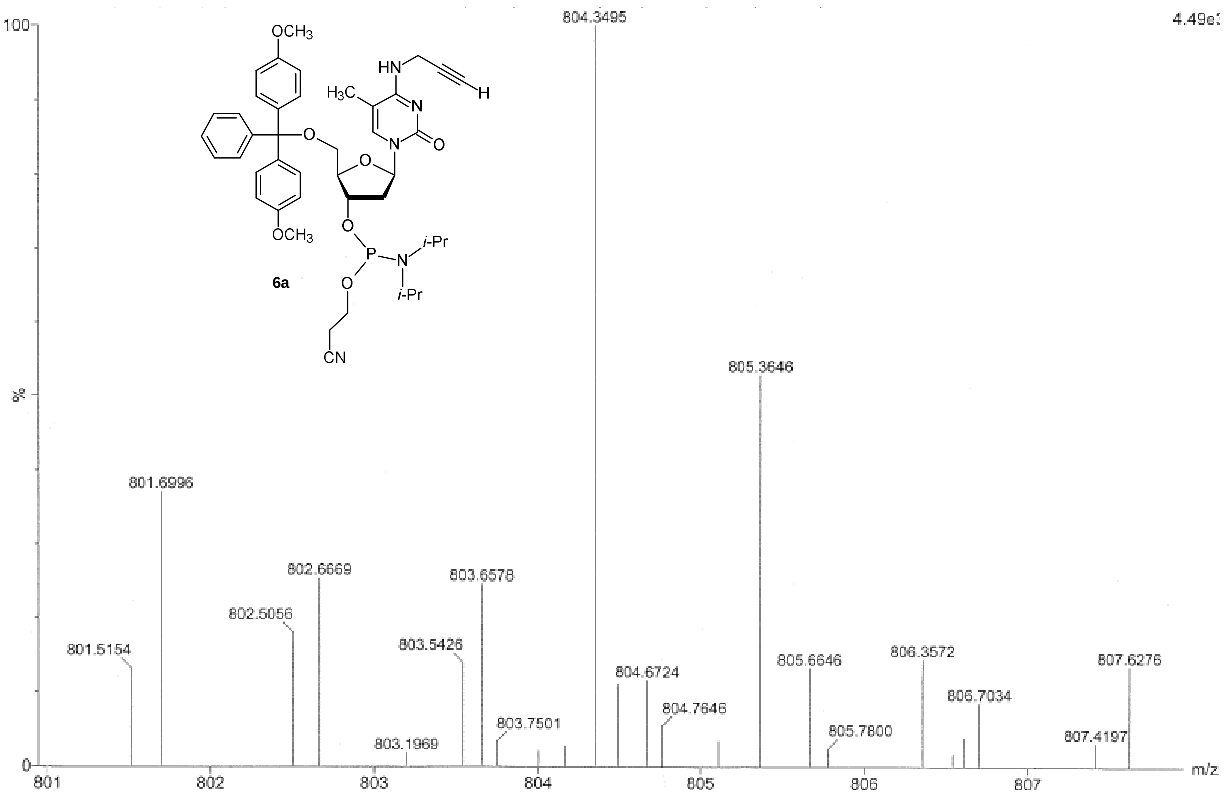

Figure S4. HR-MS (ESI) of 3´-*O*-CEP-5´-*O*-DMT-5-methyl-*N*[4]-propargyl-2´-deoxycytidine (**6a**).

Spectra of 5-Methyl-N^4-propargyl-2´-deoxycytidine (7a)

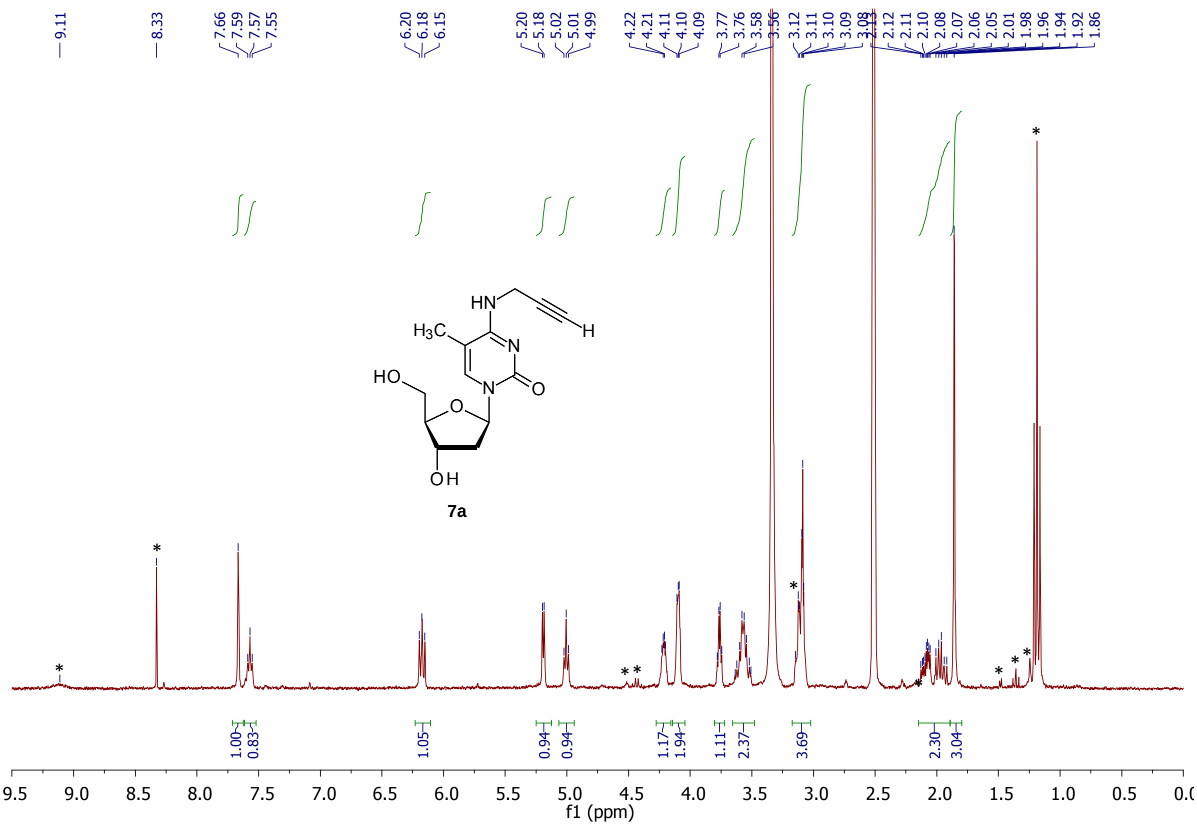

Figure S5. ^{1}H NMR (300 MHz, DMSO-d_6) of 5-methyl-N^4-propargyl-2´-deoxycytidine (**7a)**.

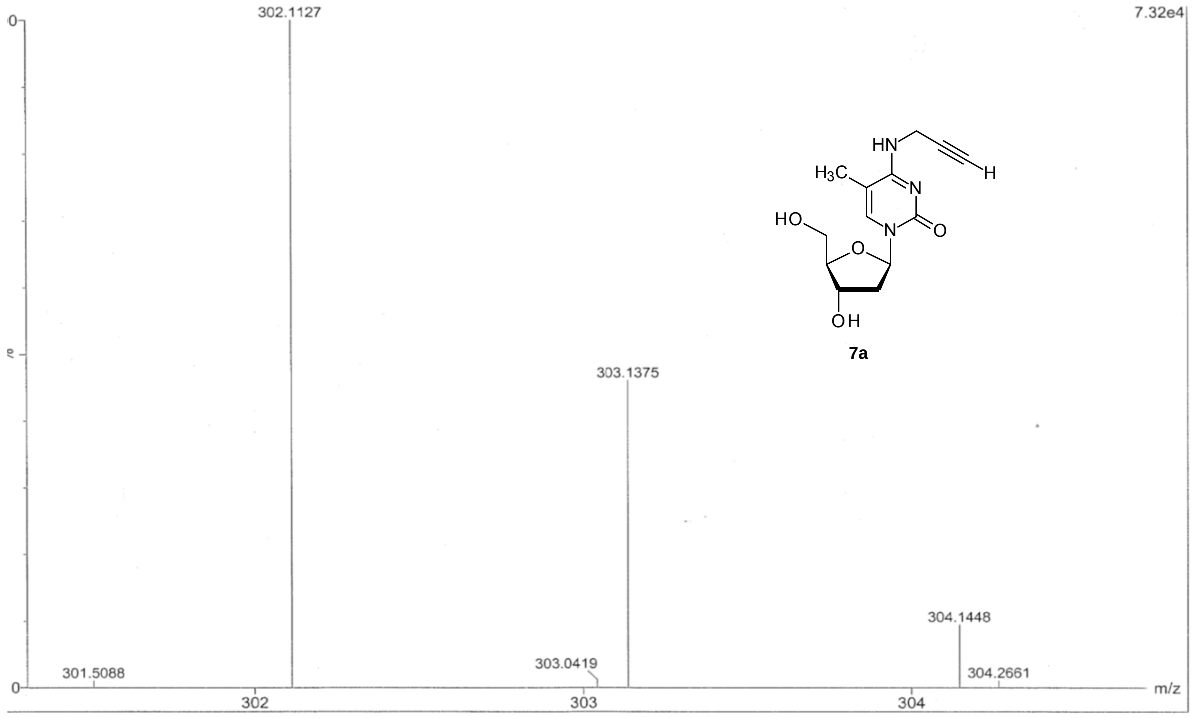

Figure S6. HR-MS (ESI) of 5-methyl-N^4-propargyl-2´-deoxycytidine (**7a**).

Temperature Dependent ^{1}H NMR Spectra of 5-Methyl-N^4-propargyl-2´-deoxycytidine (7a)

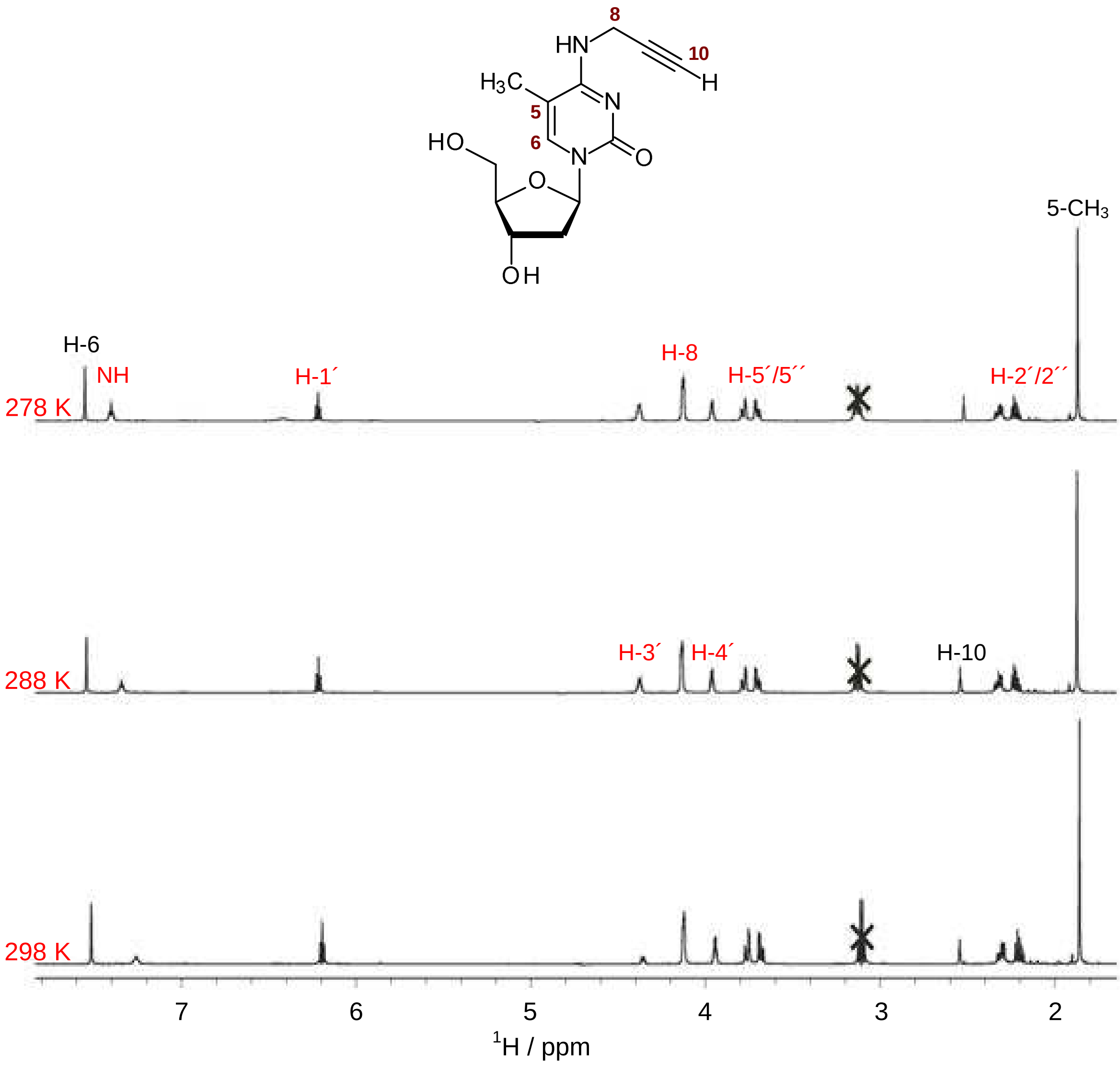

Figure S7. Temperature dependent ^{1}H NMR experiments (300 MHz, H_2O/D_2O = 9:1) of 5-methyl-N^4-propargyl-2´-deoxycytidine (**7a**) for comparison with the corresponding experiment of N^4-propargyl-2´-deoxycytidine (**7b**, see **Figure 2c**).

Spectra of 3´-*O*-CEP-5´-*O*-DMT-*N*⁴-propargyl-2´-deoxycytidine (6b)

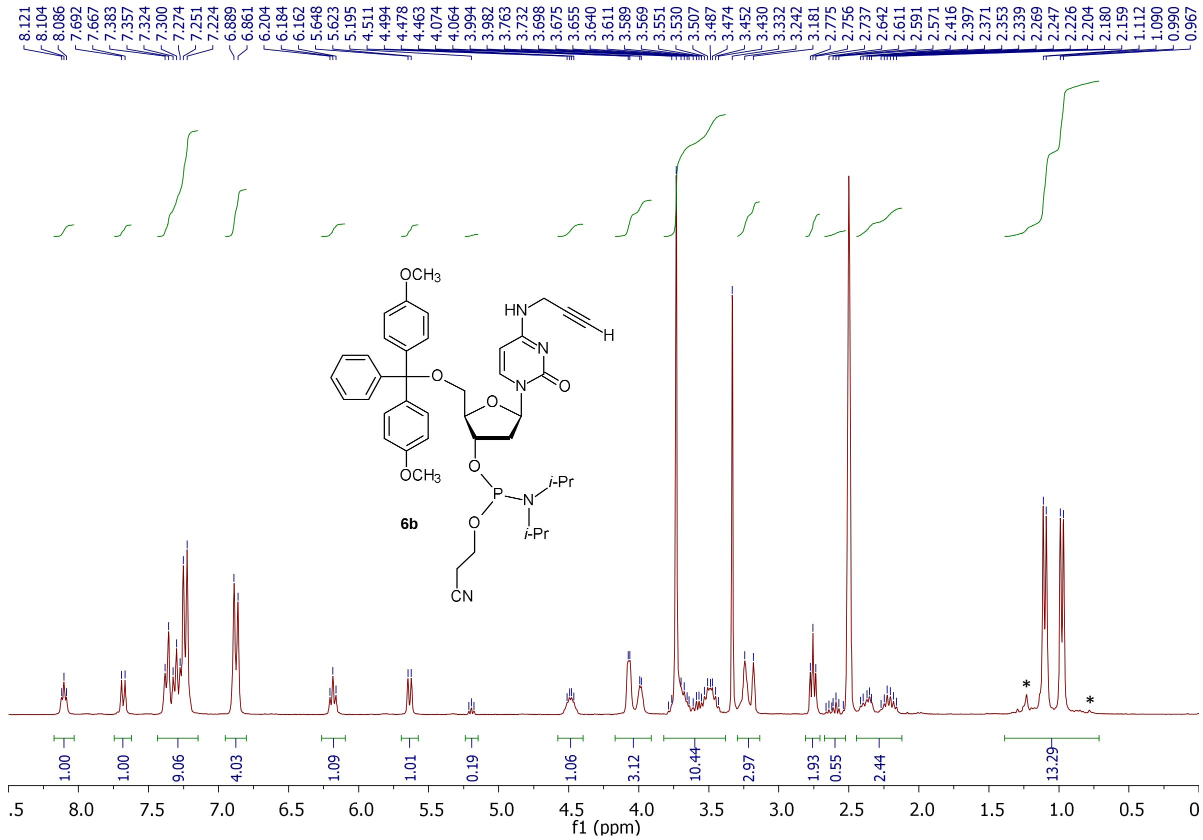

Figure S8. ^{1}H NMR (400 MHz, DMSO-d_6) of 3´-*O*-CEP-5´-*O*-DMT-*N*⁴-propargyl-2´-deoxycytidine (**6b**).

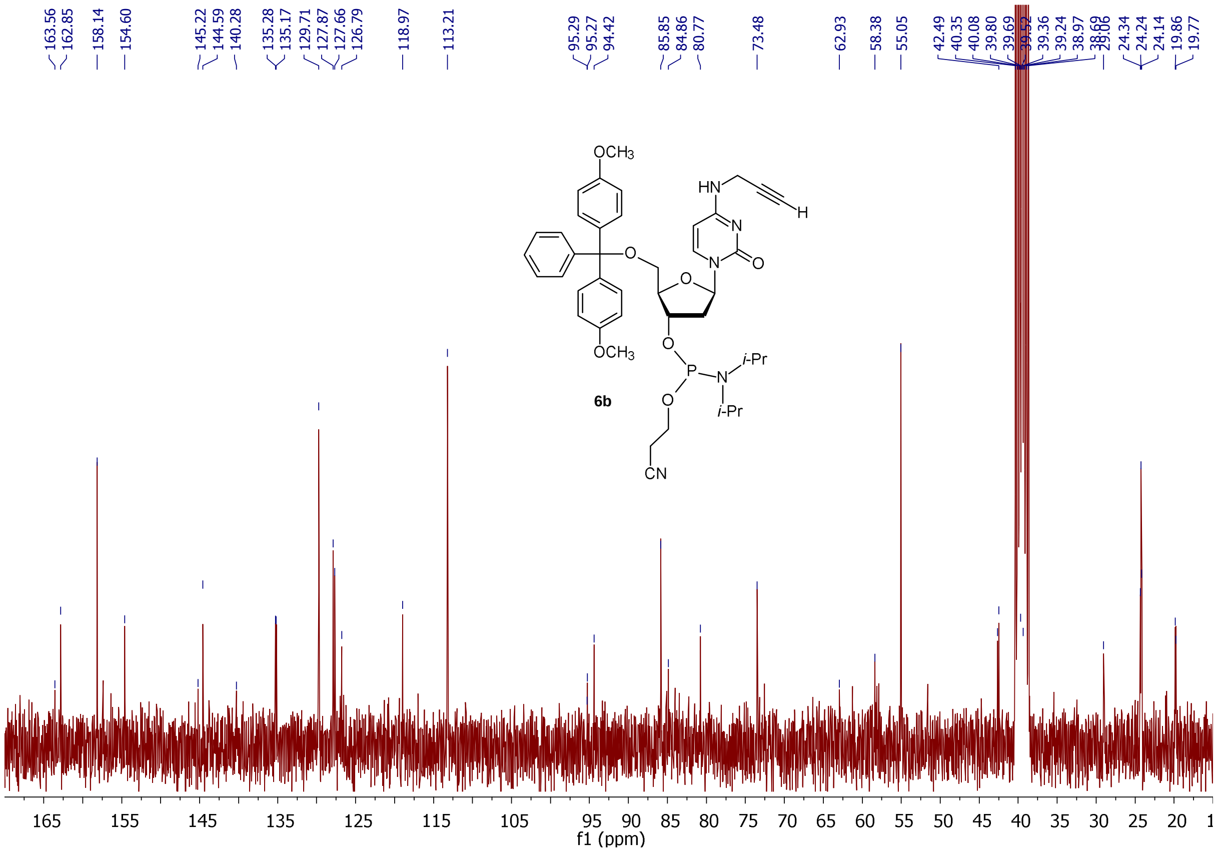

Figure S9. ^{13}C NMR (100 MHz, DMSO-d_6) of 3´-*O*-CEP-5´-*O*-DMT-*N*⁴-propargyl-2´-deoxycytidine (**6b**).

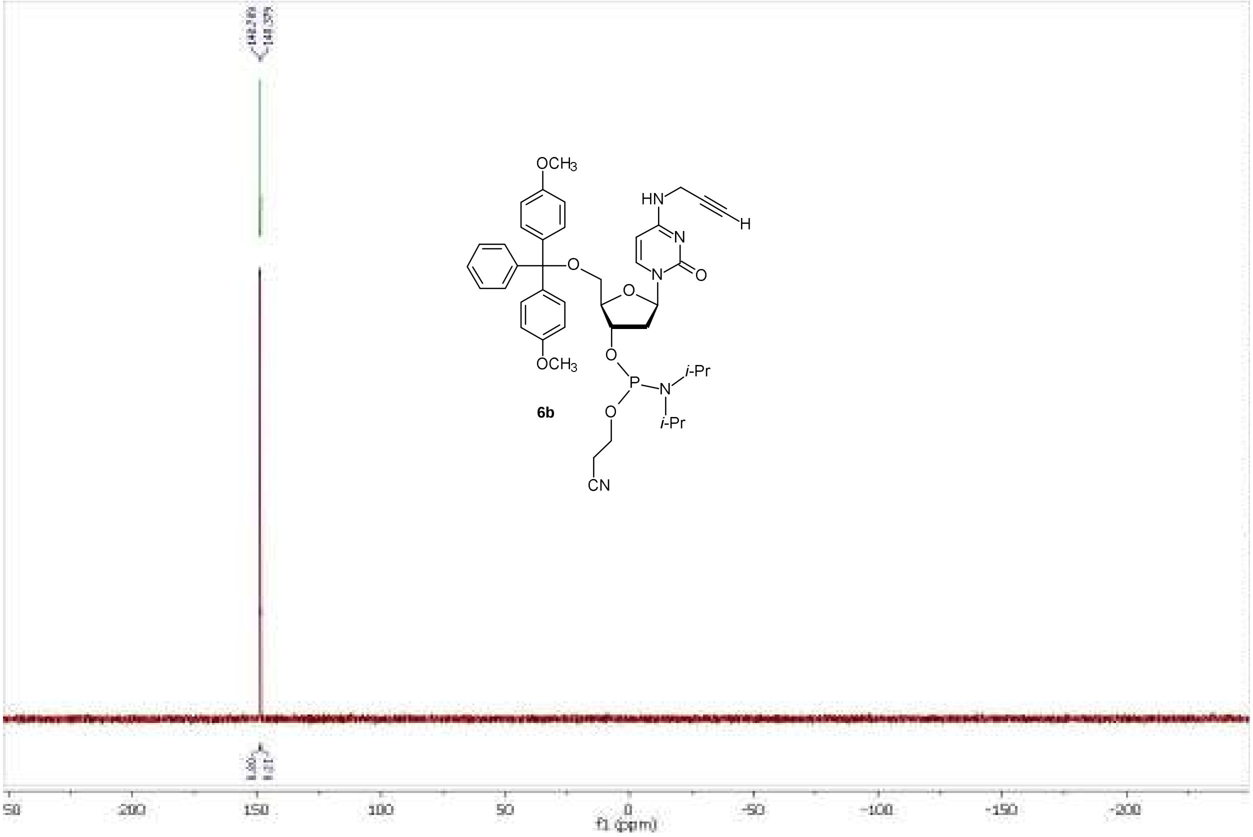

Figure S10. ^{31}P NMR (162 MHz, DMSO-d_6) of 3´-*O*-CEP-5´-*O*-DMT-*N*^{4}-propargyl-2´-deoxycytidine (**6b**).

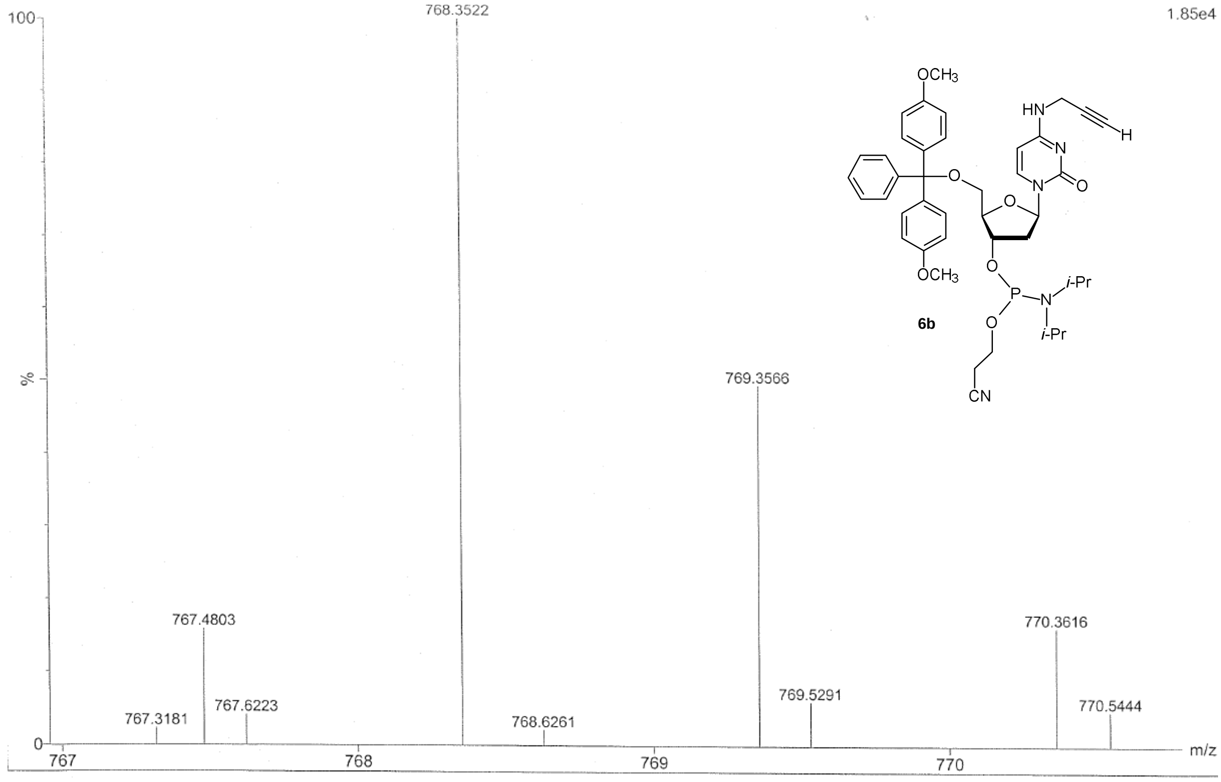

Figure S11. HR-MS (ESI) of 3´-*O*-CEP-5´-*O*-DMT-*N*^{4}-propargyl-2´-deoxycytidine (**6b**).

Spectra of *N*[4]-Propargyl-2´-deoxycytidine (**7b**)

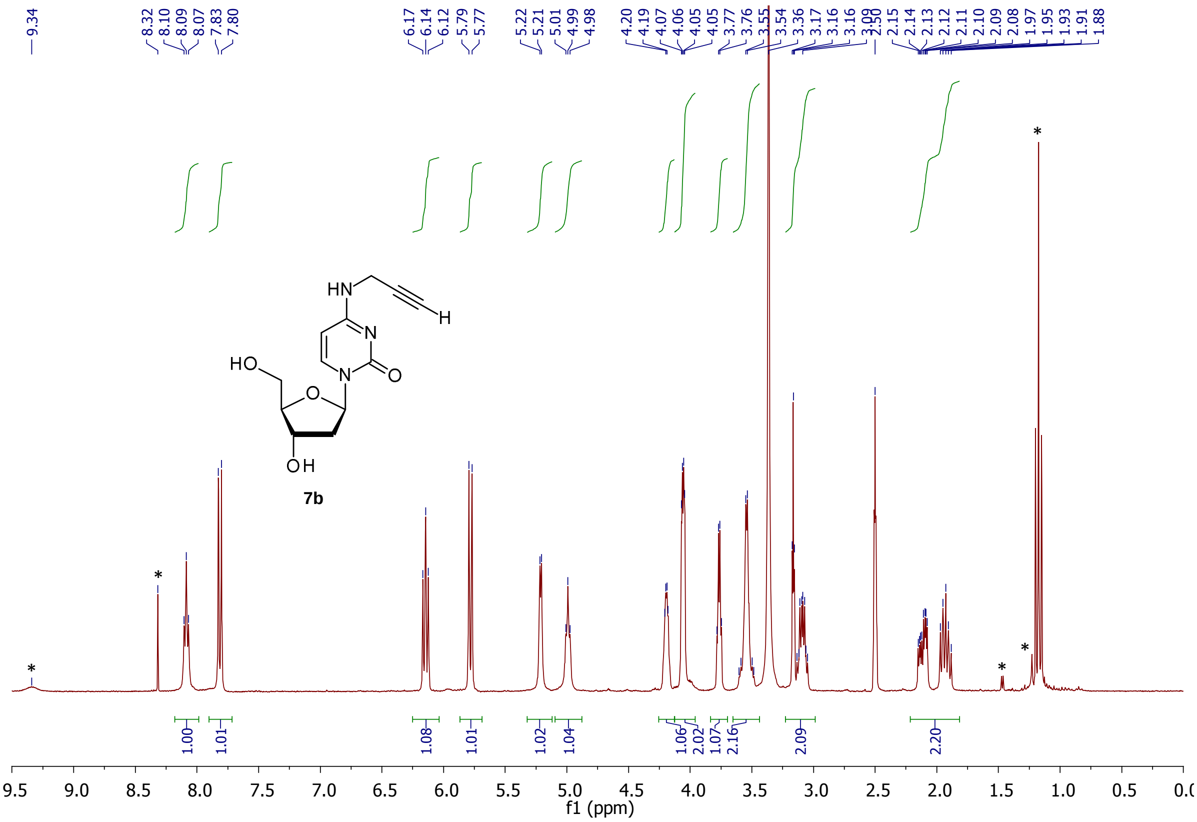

Figure S12. ^{1}H NMR (300 MHz, DMSO-d_6) of *N*[4]-propargyl-2´-deoxycytidine (**7b**).

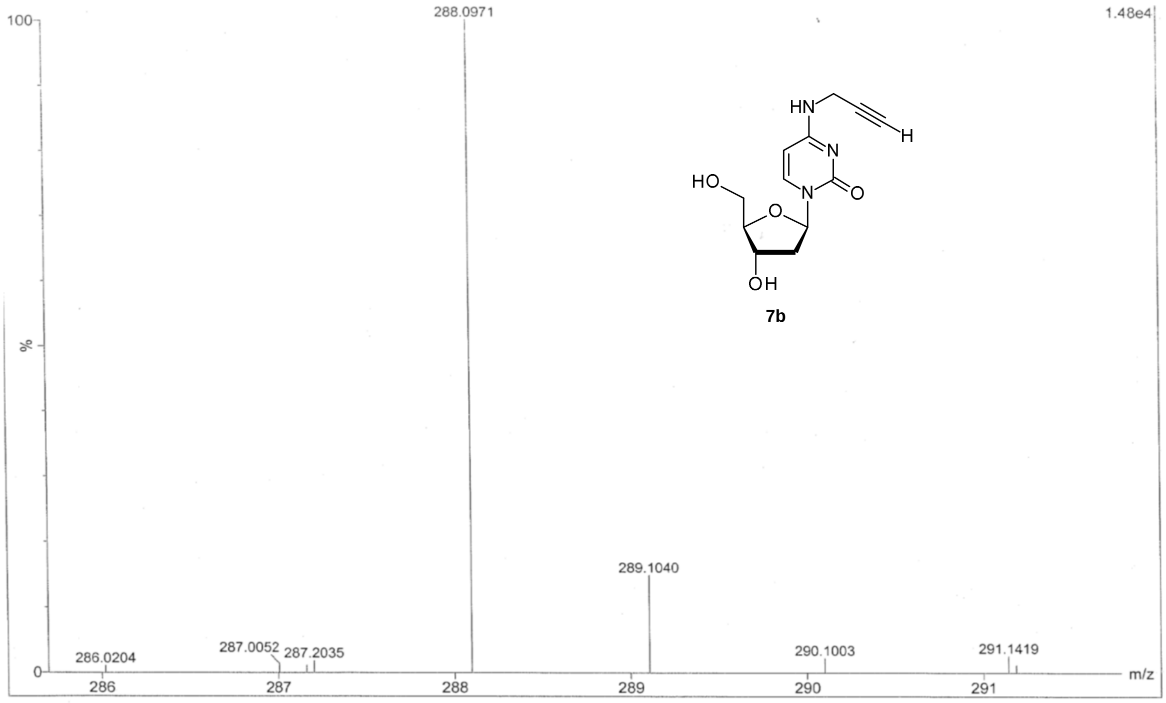

Figure S13. HR-MS (ESI) of *N*[4]-propargyl-2´-deoxycytidine (**7b**).

Temperature Dependent ROESY NMR Experiments and Arrhenius Analysis of *N*4-Propargyl-2´-deoxycytidine (7b)

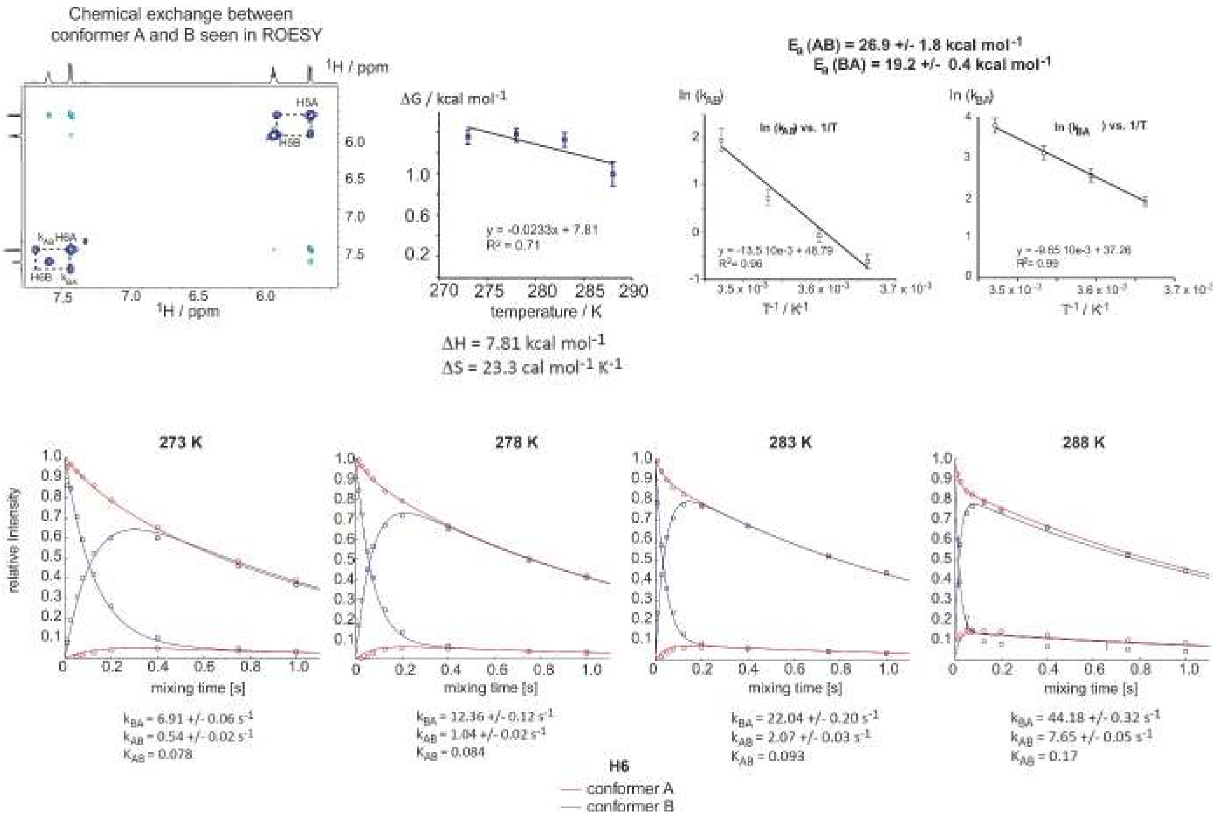

Figure S14. Temperature dependent ROESY NMR experiments (300 MHz, H_2O/D_2O = 9:1) and Arrhenius analysis of *N*4-propargyl-2´-deoxycytidine (**7b**). Details are described in the Materials and Methods section.

Oligonucleotide Synthesis and Work-up Procedure

The oligodeoxynucleotides were synthesised at a 1 µmol scale on an Expedite 8909 DNA/RNA synthesiser (ABI/PerSeptiveBiosystems) in the DMT-off mode and by using strandard ß-cyanoethyl phosphoramidite chemistry. Standard chemical procedures for deprotection and cleavage of the obtained oligonucleotides from the preloaded CPG solid support was followed, *i.e.* treatment with a 3:1 mixture of concentrated NH_3 in water/ethanol (3:1) at 55 ℃ overnight. After dr ying of the crude oligonucleotides using a SpeedVac, the samples were injected onto a Dionex DNAPac PA200 anion exchange column via HPLC to separate the full-length oligonucleotide strands from the abortion products. An elution system consisting of increasing concentrations of $NaClO_4$ in Tris buffer (pH 8) was used. The following gradient for anion exchange purification was used:

Buffer A: 20 mM Tris (pH 8)
Buffer B: 400 mM $NaClO_4$ in buffer A

TIME	% B
**0**	0
**20**	30
**25**	80
**30**	80
**35**	0

The desired oligodeoxynucleotides were obtained after desalting on Nap columns and were ready for hybridisation and melting experiments. The masses of the synthesised oligonucleotides were confirmed by MALDI-TOF mass spectra (positive mode), which were obtained from a Bruker BIFLEX III spectrometer using the matrix 3-hydroxypicolinic acid/$(NH_4)_2$-citrate.

Table S1. Sequences of the in-house synthesised oligodeoxynucleotides, showing the varying positions of modification.

* 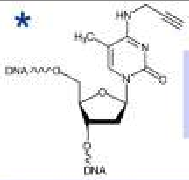	***ODN0*** 5'GCAAGCTGACCCTGAAGTTCAT3'	* 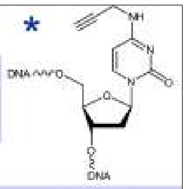
ODN2.1	5'G*AAGCTGACCCTGAAGTTCAT3'	*ODN2.2*
ODN6.1	5'GCAAG*TGACCCTGAAGTTCAT3'	*ODN6.2*
ODN10.1	5'GCAAGCTGA*CCTGAAGTTCAT3'	*ODN10.2*
ODN11.1	5'GCAAGCTGAC*CTGAAGTTCAT3'	*ODN11.2*
ODN12.1	5'GCAAGCTGACC*TGAAGTTCAT3'	*ODN12.2*
ODN20.1	5'GCAAGCTGACCCTGAAGTT*AT3'	*ODN20.2*
ODN21.1	5'GCAAGCTGACCCTGAAGTTC*T3'	*ODN21.2*

MALDI-TOF Spectra of Selected In-house Synthesised Oligodeoxynucleotides

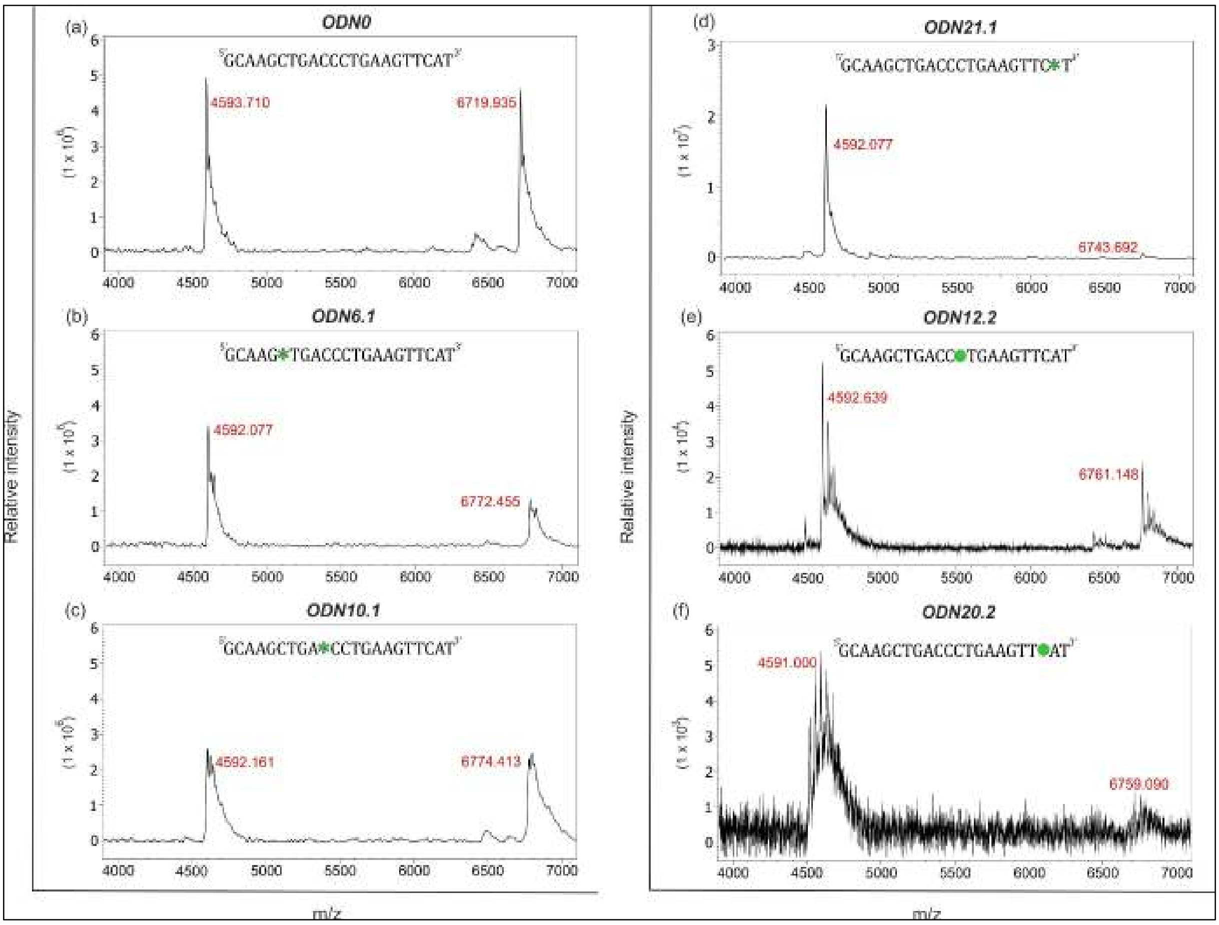

Figure S15. MALDI-TOF spectra of selected in-house synthesised oligodeoxynucleotides. A shorter DNA strand (calculated mass: 4592) was used as internal standard. (a) Unmodified strand, *ODN0* (calculated mass: 6719), (b) *ODN6.1* and (c) *ODN10.1*(calculated mass: 6771), (d) *ODN21.1*(calculated mass: 6747): * 5-methyl-N^4-propargyl-2´-deoxycytidine; (e) *ODN12.2* and (f) *ODN20.2* (calculated mass: 6757): ● N^4-propargyl-2´-deoxycytidine.

Temperature Dependent UV Absorption Melting Curves

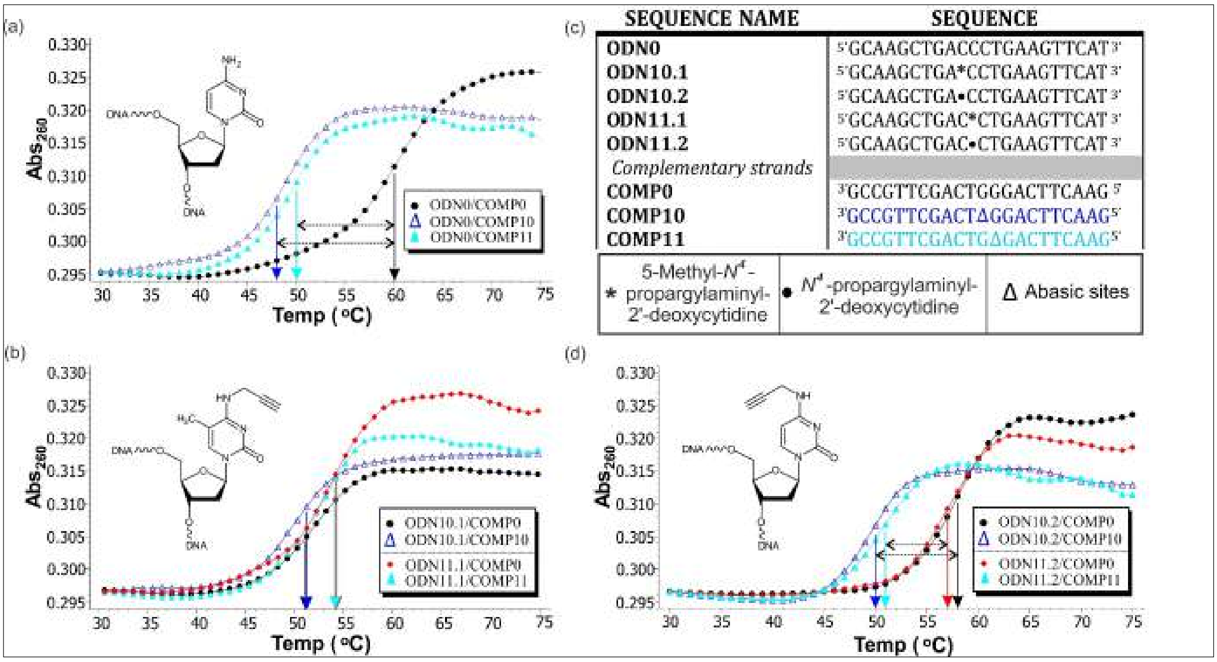

Figure S16. Temperature dependent UV-absorption melting curves, showing the differences in T_m between duplexes with and without abasic sites for DNA strands containing (a) no modifications, (b) the 5-methyl-N^4-propargyl-2´-deoxycytidine derivative and (d) the N^4-propargyl-2´-deoxycytidine derivative as modification at two varying positions. The sequences of the different DNA strands as well as the position of the modifications are indicated (c).
